# CRISPRi screens reveal a DNA methylation-mediated 3D genome dependent causal mechanism in prostate cancer

**DOI:** 10.1038/s41467-021-21867-0

**Published:** 2021-03-19

**Authors:** Musaddeque Ahmed, Fraser Soares, Ji-Han Xia, Yue Yang, Jing Li, Haiyang Guo, Peiran Su, Yijun Tian, Hyung Joo Lee, Miranda Wang, Nayeema Akhtar, Kathleen E. Houlahan, Almudena Bosch, Stanley Zhou, Parisa Mazrooei, Junjie T. Hua, Sujun Chen, Jessica Petricca, Yong Zeng, Alastair Davies, Michael Fraser, David A. Quigley, Felix Y. Feng, Paul C. Boutros, Mathieu Lupien, Amina Zoubeidi, Liang Wang, Martin J. Walsh, Ting Wang, Shancheng Ren, Gong-Hong Wei, Housheng Hansen He

**Affiliations:** 1 Princess Margaret Cancer Center/University Health Network, Toronto, ON Canada; 2 Faculty of Biochemistry and Molecular Medicine, Biocenter Oulu, University of Oulu, Oulu, Finland; 3 Changhai Hospital, Shanghai, China; 4 Department of Medical Biophysics, University of Toronto, Toronto, ON Canada; 5 Department of Tumor Biology, H. Lee Moffitt Cancer Center and Research Institute, Tampa, FL USA; 6 Department of Genetics, Washington University in St. Louis, St. Louis, MO USA; 7 Ontario Institute for Cancer Research, Toronto, ON Canada; 8 Vector Institute, Toronto, ON Canada; 9 Department of Urology, David Geffen School of Medicine, University of California, Los Angeles, Los Angeles, CA USA; 10 Department of Pharmacological Sciences, Icahn School of Medicine at Mount Sinai, New York, NY USA; 11 Department of Genetics and Genomic Sciences, Icahn School of Medicine at Mount Sinai, New York, NY USA; 12 The Vancouver Prostate Centre, Vancouver General Hospital and Department of Urologic Sciences, The University of British Columbia, Vancouver, BC Canada; 13 Helen Diller Family Comprehensive Cancer Center, University of California at San Francisco, San Francisco, CA USA; 14 Department of Urology, University of California at San Francisco, San Francisco, CA USA; 15 Department of Medicine, University of California at San Francisco, San Francisco, CA USA; 16 Department of Radiation Oncology, University of California at San Francisco, San Francisco, CA USA; 17 Department of Human Genetics, University of California, Los Angeles, Los Angeles, CA USA; 18 Jonsson Comprehensive Cancer Center, David Geffen School of Medicine, University of California, Los Angeles, Los Angeles, CA USA; 19 Institute for Precision Health, University of California, Los Angeles, Los Angeles, CA USA; 20 Fudan University Shanghai Cancer Center, School of Basic Medical Sciences, Department of Biochemistry and Molecular Biology, Shanghai Medical College of Fudan University, Shanghai, China

**Keywords:** Cancer epigenetics, Prostate cancer, Cancer epigenetics, Chromatin, DNA methylation

## Abstract

Prostate cancer (PCa) risk-associated SNPs are enriched in noncoding cis-regulatory elements (rCREs), yet their modi operandi and clinical impact remain elusive. Here, we perform CRISPRi screens of 260 rCREs in PCa cell lines. We find that rCREs harboring high risk SNPs are more essential for cell proliferation and H3K27ac occupancy is a strong indicator of essentiality. We also show that cell-line-specific essential rCREs are enriched in the 8q24.21 region, with the rs11986220-containing rCRE regulating *MYC* and *PVT1* expression, cell proliferation and tumorigenesis in a cell-line-specific manner, depending on DNA methylation-orchestrated occupancy of a CTCF binding site in between this rCRE and the *MYC* promoter. We demonstrate that CTCF deposition at this site as measured by DNA methylation level is highly variable in prostate specimens, and observe the *MYC* eQTL in the 8q24.21 locus in individuals with low CTCF binding. Together our findings highlight a causal mechanism synergistically driven by a risk SNP and DNA methylation-mediated 3D genome architecture, advocating for the integration of genetics and epigenetics in assessing risks conferred by genetic predispositions.

## Abstract (web summary)

Prostate cancer risk-associated SNPs are enriched in noncoding CREs. Here the authors perform CRISPRi screens of CREs in prostate cancer cell lines to describe a causal mechanism synergistically driven by a risk SNP and DNA methylation-mediated 3D genome architecture.

## Introduction

Prostate Cancer (PCa) is a leading cause of cancer-related mortality in men and one of the most heritable forms of cancer^1^. Genome-wide association studies (GWAS) have identified more than 160 risk loci that harbor thousands of SNPs associated with the risk for PCa, cumulatively explaining ~28% of the familial risk for PCa^2–4^. Some of these risk loci are also associated with aggressiveness of PCa^5–8^. It is thus imperative to understand the mechanisms of how these SNPs function and to translate PCa GWAS findings to the clinic. Similar to many other cancer types, about 98% of the PCa risk SNPs are located outside of coding exons, and thus do not function through altering protein-coding sequences^4,9^. In fact, previous studies have shown that the noncoding risk variants are significantly enriched in *cis*-regulatory elements (CREs)^9–12^. Several noncoding SNPs have been identified to alter CRE functions to *cis*-modulate target gene expression^7,9,13–15^. However, systematic functional dissection of these risk SNP-containing CREs (rCREs) on a genome-wide scale remains a challenge and is essential for understanding their clinical impact.

The recent advent of CRISPR/Cas9-mediated genome editing approaches has made a systematic assessment of CREs possible by its virtue of high specificity and scalability. Several studies recently implemented this approach to functionally dissect targeted CREs^16–20^. Several variations have been developed to widen the applicability, including CRISPR interference (CRISPRi). In this technique, the Cas9 nuclease is mutated to generate catalytically dead Cas9 (dCas9) and fused with a repressor protein, such as KRAB, to functionally suppress the targeted chromatin region^21^. RNA-guided recruitment of dCas9-KRAB benefits not only from repressing chromatin regions without altering the DNA sequence but also bypassing the confounding effect of copy number alterations^21–27^.

We and other groups have previously identified hundreds of rCREs that harbor at least one PCa-associated risk SNP^7,9,13^. In this study, we aimed to evaluate the essentiality of these rCREs in PCa using CRISPRi-mediated loss-of-function screens. Our screens reveal that rCREs essential for PCa cell growth are enriched in the gene desert region of 8q24.21. The 8q24.21 region was one of the first functionally dissected risk loci in PCa, which contains many PCa risk SNPs that cumulatively explain 25% of the familial risk for PCa^28^. The *MYC* oncogene in this region is highly expressed and frequently amplified in PCa, making it a prime candidate gene to be linked with inherited PCa risk^9,29,30^. Despite the large number of risk SNPs and rCREs in 8q24.21, studies have failed to identify the clear association of any genotype with *MYC* expression^31–33^, although several reports indicated physical interaction between rCREs and *MYC* promoter in cell line models^10,33,34^. One of the most essential rCRE identified in our screens regulates *MYC* and harbors the risk SNP rs11986220, which confers high odds ratio (OR) for PCa risk^13,35,36^. Here, we find that the interaction between this rCRE and *MYC* promoter is disrupted by CTCF deposition at a site about 10 Kb upstream of *MYC* transcription start site (TSS), and this CTCF deposition is DNA methylation dependent. CTCF is a key regulator of the three-dimensional (3D) genome architecture^37–43^, and disruption of CTCF-mediated 3D chromatin interactions may lead to dysregulation of neighboring genes in various cancer types^38,44–48^. In addition to disrupting the CRE function, our study unveils that the CTCF deposition at this locus also reduces the causal effect of rs11986220. This emphasizes the complexity of the 8q24.21 region, which is subjected to multiple CTCF-mediated looping, and indicates that the *MYC*-genotype associations are perhaps heavily 3D genome dependent.

## Results

### CRISPRi screens with tiling sgRNAs identify essential rCREs in PCa

**Fig. 1:**
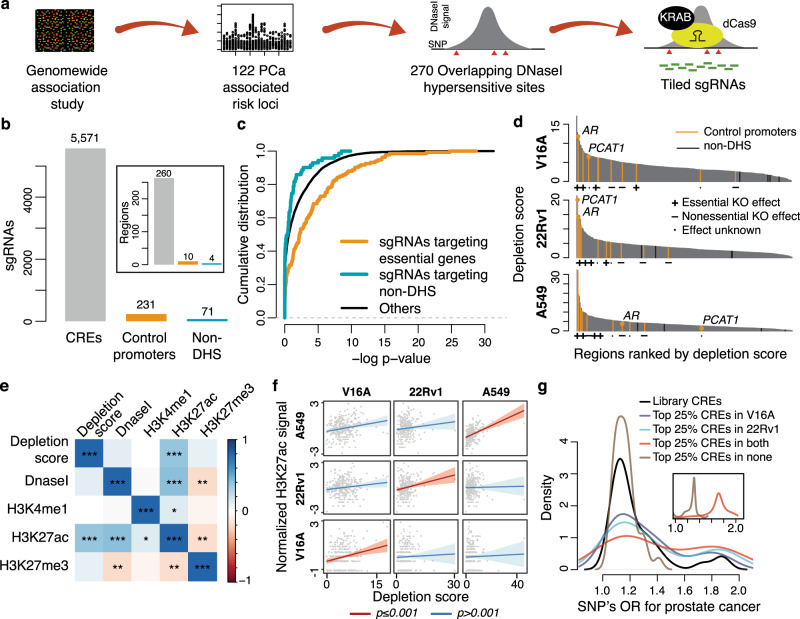
CRISPRi screening of prostate cancer risk CREs. **a** Schematic of rCRE selection and sgRNA design. **b** Distribution of sgRNAs targeting rCREs or control regions. The barplot in the inset indicates the number of regions in the library. **c** The cumulative distribution of depletion p-values of sgRNA targeting control promoter regions that are essential for growth in Achilles DepMap project (orange), sgRNAs targeting non-DNaseI hypersensitive sites (green), and sgRNAs targeting rCREs (black). Depletion *p* values were estimated using the tool MAGeCK (see ‘Methods’). **d** rCREs ranked by their depletion scores in three cell lines. Promoters of two prostate-specific oncogenes, *AR* and *PCAT1*, are labeled. The panels below the plots indicate the knockout effects of the control genes on respective cell growth as observed in Achilles DepMap project. **e** Correlation of LNCaP ChIP-seq signals of several histone marks with the depletion score in LNCaP-derived V16A cells. *P* values are estimated using Spearman’s correlation test. The colors of the box correspond to the correlation coefficient and the * corresponds to the statistical significance of correlation test. *, *p* ≤ 0.05; **, *p* ≤ 0.01; ***, *p* ≤ 0.005. **f** Linear regression between H3K27ac ChIP-seq signals and CRISPRi depletion score in cell line-specific manner. The solid lines denote the best fit for the regression model and the shaded areas denote 95% confidence interval. *P* value is calculated using linear regression analysis. **g** Distribution of odds ratio for PCa conferred by the risk SNPs within library rCREs. The black line denotes OR of risk SNPs in rCREs in the library; the colored lines denote top 25% rCREs when ranked by their depletion scores in PCa cell lines. The inset plot demonstrates the OR distribution by risk SNPs in bottom 75% rCREs (brown) and top 25% rCREs in both V16A and 22Rv1 cells (red) normalized by the overall OR distribution of all library risk SNPs. The inset axes are the same as the main plot axes.

Despite possessing defined chromatin characteristics, CREs function in multifaceted mechanisms that makes the systematic identification of core functional regions in CREs a major obstacle. Chromatin accessibility and histone modification data are often exploited to identify CREs, but these data lack the capacity to pinpoint the functional sequences in CREs, which makes them a difficult target for designing short guide RNAs (sgRNAs) in genome editing techniques such as CRISPR/Cas9^49^. To overcome this, we took an approach to tile the DNase I hypersensitive (DHS) region with sgRNAs for any particular CRE. Previously, we performed an integrative multi-omic analysis and identified 270 PCa rCREs that harbor at least one PCa risk SNP^9^. Here, we developed an algorithm to design tiling sgRNAs within each rCRE using criteria as previously described^50–53^, and selected equidistantly spaced sgRNAs to achieve maximum coverage (see ‘Methods’) (Fig. 1a). We successfully designed sgRNAs for 260 rCREs with an average of five sgRNA per 100 bp DNA (Supplementary Fig. 1a), resulting in a library of 5873 sgRNAs that tile these rCREs along with ten control promoter and four DNase I insensitive regions **(**Fig. 1b**;** Supplementary Data 1). The library was packaged into a lentiviral vector, transduced into cells stably expressing dCas9-KRAB fusion protein, and selected with puromycin. We performed the experiment in two PCa cell lines—LNCaP derived V16A, and 22Rv1 cells, each with two replicates. The population of cells expressing the library was harvested at day 0, and then again at day 16 under standard culturing conditions. Genomic DNA was extracted from harvested cells and the frequency of each sgRNA in each sample was analyzed using high throughput sequencing. The sgRNA counts at each time point were strongly correlated between the replicates in both cell lines (Supplementary Figs. 1b, c), suggesting high reproducibility of the screens.

The degree of essentiality (measures as “depletion score”, see ‘Methods’) of a locus was estimated by calculating the level of depletion of sgRNAs in day 16 compared to day 0. The cumulative depletion of sgRNAs targeting control promoter regions of essential genes (as identified in Achilles CRISPR/Cas9 screens^25,54^) was much higher compared to the 71 sgRNAs targeting randomly selected DNase I insensitive regions (Fig. 1c). In parallel to the V16A and 22Rv1 cell lines, we performed similar screens in a non-small cell lung cancer cell line, A549, to gauge the cancer-type specificity of the PCa rCREs. Across all three cell lines, when all the regions were ranked in order of their depletion scores, the control promoters with high depletion scores are of genes that have high essentiality scores in Achilles CRISPR-Cas9 screens in respective cells (Fig. 1d and Supplementary Figs. 1d–f**;** Supplementary Data 2, 3)^25^. These control promoters are also distinctly separated from the DNase I insensitive sites, validating the efficiency of our screens (Fig. 1d). When the promoters are ranked in order of their depletion score in A549 cells, four out of the top five promoters, *RPS8, POLR2D, POLR1C*, and *U2AF1*, are of housekeeping genes that are also essential in Achilles CRISPR-Cas9 screens in A549 cells (Fig. 1d and S1f**;** Supplementary Data 4). The library control promoters of genes specifically associated with PCa biology, *AR* and *PCAT1*, are among the top 18 and three most depleted regions in V16A and 22Rv1 cell lines, respectively, but are not ranked within the top 65 regions in A549 cells (Fig. 1d). In fact, the least depleted control promoter in A549 is *PCAT1* (Fig. 1d), which is specifically expressed in PCa^55^. When the statistical significance of depletion of sgRNAs targeting rCREs in all three cell lines was compared, the two PCa cell lines had a similar distribution of *p* values distinct from that in A549 cells (*p* < 0.0001; Kolmogorov–Smirnov test) (Supplementary Fig. 1g). Overall, these data suggest that the CRISPRi screens were able to successfully identify regions essential for cellular proliferation.

### Highly essential rCREs harbor SNPs conferring higher risk for PCa

CREs are typically defined by epigenetic modifications of nearby histone molecules^56^. Hence it is important to determine if essential rCREs can be distinguished from nonessential rCREs by epigenetic marks. We correlated the depletion scores from CRISPRi screens in the LNCaP-derived V16A cells with abundance of several histone modifications as identified by ChIP-seq assays in the LNCaP cell line. The depletion scores strongly correlated with H3K27ac signal that is typically associated with the active state of a chromatin region (Fig. 1e)^57^, but not with H3K4me1 signal, which is a typical mark for enhancer regions irrespective of activity status^58^. When comparing the depletion scores of rCREs with H3K27ac ChIP-seq signal in all three cell lines, we observed a strong correlation between H3K27ac signal and depletion scores in a cell line-specific manner (Fig. 1f).

Each rCRE in our library harbors at least one PCa risk SNP. Since the genetic risk conferred by each risk SNP varies^9,13,59^, we thus examined the association between risk OR and essentiality of rCREs. When we separate the library rCREs into most depleted in any PCa cell line and most depleted in both cell lines, we observe that the SNPs in the most depleted rCREs confer progressively higher OR for PCa (Fig. 1g). Importantly, the median OR conferred by SNPs in most depleted rCREs in both PCa cell lines is significantly higher than the median OR conferred by SNPs in less depleted rCREs (Fig. 1g inset). These data indicate that SNPs posing a higher risk for PCa tend to be located in CREs that are highly essential for PCa growth.

### The gene desert region of 8q24.21 is enriched with essential rCREs

**Fig. 2:**
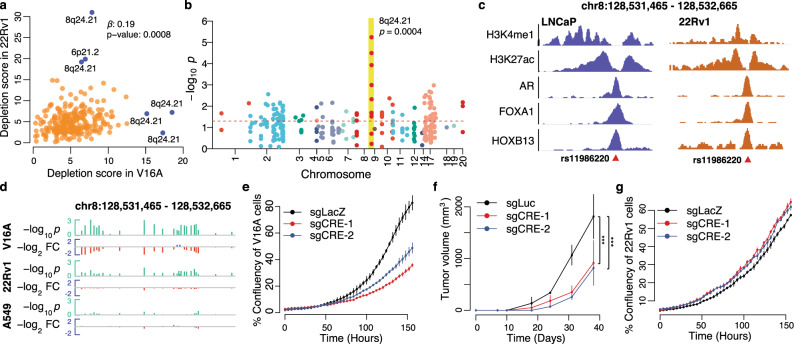
The essential rCREs are enriched in 8q24.21 region. **a** Depletion score of rCREs in V16A and 22Rv1 cells. The blue points indicate the outlier rCREs in linear regression between the cell lines. The regression coefficient, β, and the *p* value are calculated using linear regression analysis between the depletion scores after removing the six outliers. **b** The essential rCREs are overrepresented in the 8q24.21 region in V16A cells (*p* = 0.0004, Chi-sq test). Each circle denotes a library rCRE. The size of the circle is relative to the depletion fold change. See Supplementary Fig. 2 for other cell lines. **c** ChIP-seq signals of histone modifications and three important transcription factors in the rCRE region of chr8:128531465–128532665 in LNCaP and 22Rv1 cells. Risk SNP rs11986220 is located close to the center of transcription factors binding site. **d** Overview of *p* value and fold change at day 16 compared to day 0 of the individual sliding windows targeting the rCRE chr8:128531465–128532665. The green bars indicate –log_2_ *p* values; the red bar indicates fold change of sgRNAs in day 16 compared to day 0. FC fold change. Depletion *p* values and fold changes were estimated using the tool MAGeCK (see ‘Methods’). **e** Growth of V16A cells in vitro upon suppression of chr8:128531465–128532665 by two independent sgRNAs using dCas9-KRAB system. Data are represented as Mean ± s.d. (*n* = 2). **f** Tumor growth in a V16A-inoculated mouse xenograft upon injection of respective sgRNAs. Data are represented as Mean ± s.d. (*n* = 3). *P* values were estimated using ANOVA test. *** denotes a *p* value of 0.007. **g** Growth of 22Rv1 cells upon suppression of this rCRE using the same sgRNAs by dCas9-KRAB system. Data are represented as Mean ± s.d. (*n* = 2). Source data are provided as a Source Data file.

Despite being two distinct PCa cell lines, the depletion scores in the CRISPRi screens in V16A and 22Rv1 were positively associated (*p* = 0.0008, linear regression; *p* = 0.0008, Pearson’s correlation test; Combined *p* = 9.3e–69, Empirical Brown’s method) (Fig. 2a). The overlap of essential rCREs between the two PCa cell lines is higher than that with A549 (Supplementary Fig. 1g inset). Applying an outlier test method (see ‘Methods’), we identified six rCREs that have differential essentiality between the two cell lines (Fig. 2a, marked in blue), and five of them are located in the gene desert region of 8q24.21. As a matter of fact, our CRISPRi screen data reveals that 8q24.21 region is significantly overrepresented by essential rCREs (*p* value < 0.05) in PCa cell lines but not in A549 cells (Fig. 2b and Supplementary Figs. 2a–c). The 8q24.21 region is one of the first dissected risk loci associated with PCa and is in the vicinity of important oncogenes including *MYC* and *PVT1*^60–64^. Our screens demonstrate that eight out of the ten rCREs in this region are essential in at least one PCa cell line, whereas none of them are essential in A549 (Supplementary Fig. 2d). All of the eight essential rCREs are marked with H3K27ac histone modification in LNCaP cells, with six of them being also marked with H3K4me1 modification (Supplementary Fig. 2e).

The only differential essential rCRE outside of 8q24.21 is located in 6p21.2 (chr6:41514080–41514480, FOXP4 promoter), which has a depletion score significantly higher in 22Rv1 than in V16A (Fig. 2a). Further analysis revealed that this rCRE is abundantly marked with H3K27ac modification only in 22Rv1 but not in LNCaP cells (Supplementary Fig. 2f). Among the two rCREs in the 8q24.21 region with significantly higher depletion scores in 22Rv1 compared to V16A cells, the one at chr8:128112295–128112695 has a FOXA1 binding specific to 22Rv1 cells (Supplementary Fig. 2g). The other rCRE at chr8:12802795–128028315 is located in the intron (~3 Kb downstream of TSS) of *PCAT1* (Supplementary Fig. 2h), the promoter of which confers higher essentiality in 22Rv1 (ranked 2nd amongst all screened regions) than V16A (ranked 18) cells (Fig. 1d). Among the three rCREs conferring higher essentialities in V16A compared to 22Rv1 cells, two are located closely in the region of chr8:128103955–128105195, which harbors a strong AR binding site specifically in LNCaP but not in 22Rv1 cells (Supplementary Fig. 2g). The rCRE chr8:128531465–128532265 has similar H3K27ac level, as well as binding of multiple transcription factors, including AR, FOXA1, and HOXB13 in both cell lines (Fig. 2c); hence, we focus on this rCRE for further investigation.

### rs11986220-containing rCRE promotes cell line-specific proliferation and transcriptional changes via modulating *MYC* expression

There are 24 100 bp sliding windows (see ‘Methods’) containing at least two sgRNAs targeting the chr8:128531465–128532265 rCRE, and most of them show strong depletion in V16A but not in 22Rv1 or A549 cells (Fig. 2d). We performed validation experiments by targeting this rCRE using dCas9-KRAB complex guided by two independent sgRNAs and measured the cell growth rate in vitro. Both sgRNAs resulted in a decrease in the proliferation of V16A cells (Fig. 2e). To further investigate the effect of this rCRE in vivo, we injected mice with V16A cells stably expressing dCas9-KRAB complex along with sgRNAs against the rCRE or* Luc* control. A marked decrease in tumor growth was observed upon repression of this rCRE (Fig. 2f). Importantly, consistent with our screen results in 22Rv1 cells, these sgRNAs did not cause an obvious effect on the proliferation of 22Rv1 cells (Fig. 2g).

**Fig. 3:**
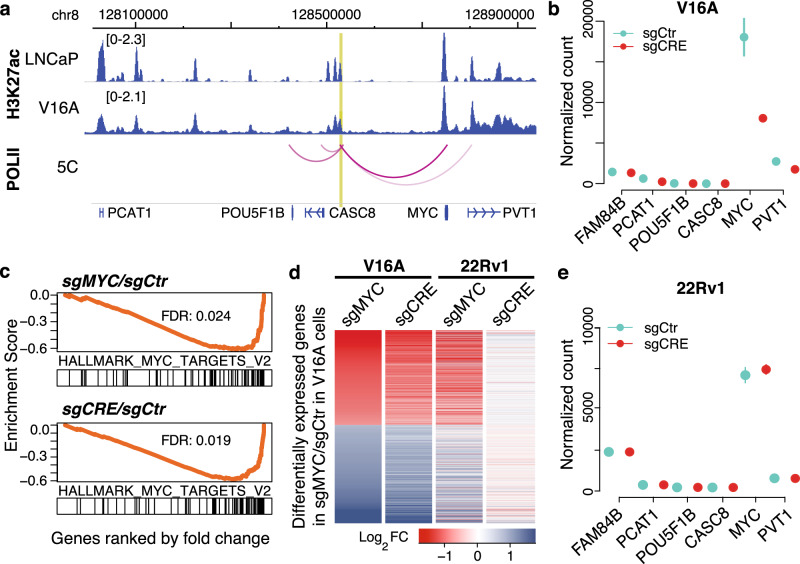
The rCRE chr8:128531465–128532265 regulates *MYC* in V16A but not in 22Rv1 cells. **a** The top two tracks demonstrate the H3K27ac ChIP-seq signal in LNCaP and V16A cells. The arc track represents the interactions between this rCRE and neighboring promoter regions as determined by ENCODE POLII 5C data in LNCaP cells. The intensity of the arc color represents the interaction strength. The bottom track represents the RefSeq gene annotation. The chromosomal positions are of the genome assembly Hg19. **b** Expression of neighboring genes as determined by RNA-seq upon repression of the rCRE chr8:128531465–-128532665 by dCas9-KRAB in V16A cells. The data are shown in mean ± s.d. (*n* = 2). Source data are provided as a Source Data file. **c** Gene set enrichment analysis shows that the hallmark MYC target genes (MSigDB H collection) are most overrepresented among the genes downregulated by repression of the *MYC* promoter (upper panel) or rs11986220-CRE (lower panel). See Supplementary Fig. 3 for overall gene set enrichment analysis. **d** Changes in transcriptome-wide gene expression upon repression of the rCRE (by sgCRE) and the *MYC* promoter region (by sgMYC) in V16A and 22Rv1 cells. Only the genes differentially expressed (FDR < 0.1 and fold change >1.5, negative binomial test) upon *MYC* promoter repression (sgMYC) in dCas9-KRAB V16A cells are shown. The genes are sorted by fold change in V16A cells. FC fold change. **e** Expression of neighboring genes as determined by RNA-seq upon repression of the rCRE by dCas9-KRAB in 22Rv1 cells. The data are shown in mean ± s.d. (*n* = 2). Source data are provided as a Source Data file.

This rCRE harbors two PCa-associated risk SNPs—rs11986220 and rs10090154 (Supplementary Fig. 3a**)** that are polymorphic for A/T and T/C alleles, respectively. These SNPs are in high linkage disequilibrium in major ethnic populations (Supplementary Fig. 3a**)**^13,36^. Several genome-wide association analyses have found the minor allele A of rs11986220, or T of rs10090154, to be highly associated with PCa risk across multiple ethnic populations, conferring OR of 1.19–3.45^13,35^. In LNCaP and 22Rv1 cells, this rCRE is marked by H3K27ac and has a strong binding of AR, FOXA1, and HOXB13, all of which are critical transcription factors for PCa biology (Fig. 2c). A similar level of H3K27ac modification was also observed in V16A cells (Fig. 3a). Since the SNP rs11986220 is located near the center of the binding sites (Supplementary Fig. 3a), we used IntraGenomic Replicates analysis to predict the effect of the genotype of this SNP on transcription factors binding^15^. Consistent with previous reports^13^, the risk allele A of rs11986220 is associated with a significantly higher level of FOXA1 binding (Supplementary Fig. 3b). Furthermore, among the cancer cell lines in ENCODE, this chromatin region is accessible only in PCa cell line (Supplementary Fig. 3c). The specificity of this rCRE to the prostate tumor and its activation by androgen^13^ emphasize the importance of this rCRE in prostate transformation.

Next, we sought to identify the underlying functional mechanism of the rs11986220-containing rCRE. Chromosome Conformation Capture Carbon Copy (5C) anchoring at Pol II binding sites in LNCaP cells revealed that this rCRE interacts with four nearby genes, showing the strongest interaction with the *MYC* promoter (Fig. 3a). Consistent with the 5C data, a Chromosome Conformation Capture (3C) analysis also detected the interaction between this rCRE and *MYC* promoter in LNCaP cells^33^. This indicates that this rCRE may function as an *MYC* enhancer in PCa. To validate, we designed pairs of sgRNAs each targeting the rs11986220-containing rCRE, two negative controls, and the *MYC* promoter (Supplementary Fig. 3d**;** Supplementary Data 5). When transduced into V16A cells stably expressing dCas9-KRAB complex, sgRNAs targeting the rCRE significantly reduced the mRNA expression of *MYC* (Supplementary Fig. 3e). To assess the effect of this rCRE on the transcriptome, we performed RNA-seq in these cells upon repression of the rCRE (sgCRE) and *MYC* promoter (sg*MYC*). Amongst the genes in the vicinity, only *MYC* and *PVT1* expression was markedly reduced (fold change = 0.49 and 0.7, respectively) upon repression of this rCRE in V16A cells (Fig. 3b). Gene set enrichment analysis identified the MYC-regulated gene set to be most enriched amongst sg*MYC* target genes. The same MYC-regulated gene set was also the most enriched amongst sgCRE target genes (Fig. 3c and Supplementary Figs. 3f, g). At the transcriptome level, 912 genes were found differentially expressed upon suppression of the rs11986220-containing CRE, 67% of which were also found differentially expressed upon suppression of *MYC* promoter (Supplementary Fig. 3h). The effects of the rs11986220-containing rCRE and *MYC* promoter repression on the RNA expression of target genes were strongly correlated (Pearson’s correlation coefficient = 0.94, *p* = 2.2E–16) in V16A cells, indicating this rCRE mainly function through regulating *MYC* transcription (Fig. 3d).

In contrast to V16A cells, the rs11986220-containing rCRE is not found to be essential in 22Rv1 cells (Fig. 2g). However, this rCRE exhibits chromatin occupancy characteristic in 22Rv1 cells with AR, FOXA1, and HOXB13 binding and marked by H3K27ac and H3K4me1 modifications similar to that in LNCaP cells (Fig. 2c). Since this rCRE primarily regulates *MYC*, its non-essentiality in the 22Rv1 cells could be explained if *MYC* was not essential for 22Rv1 cell growth; however, repression of *MYC* significantly inhibits proliferation of 22Rv1 cells (Supplementary Fig. 3i). An alternative explanation is that the rs11986220-containing rCRE regulates some other genes that are not essential for the growth of 22Rv1 cells. To test that, we performed RNA-seq in 22Rv1 cells upon repression of this rCRE and *MYC* promoter using the same guide RNAs as used in V16A cells. While the effect of sg*MYC* on RNA expression of the target genes are similar between 22Rv1 and V16A cells, sgCRE treatment did not significantly alter the expression of any genes in 22Rv1 cells (Fig. 3d, e and Supplementary Fig. 3j). All these data indicate that despite having similar epigenetic characteristics as in LNCaP cells, the regulation on *MYC* expression by this rCRE is somehow lost in 22Rv1 cells.

### CTCF binding near *MYC* mediates rCRE and *MYC* promoter interaction

**Fig. 4:**
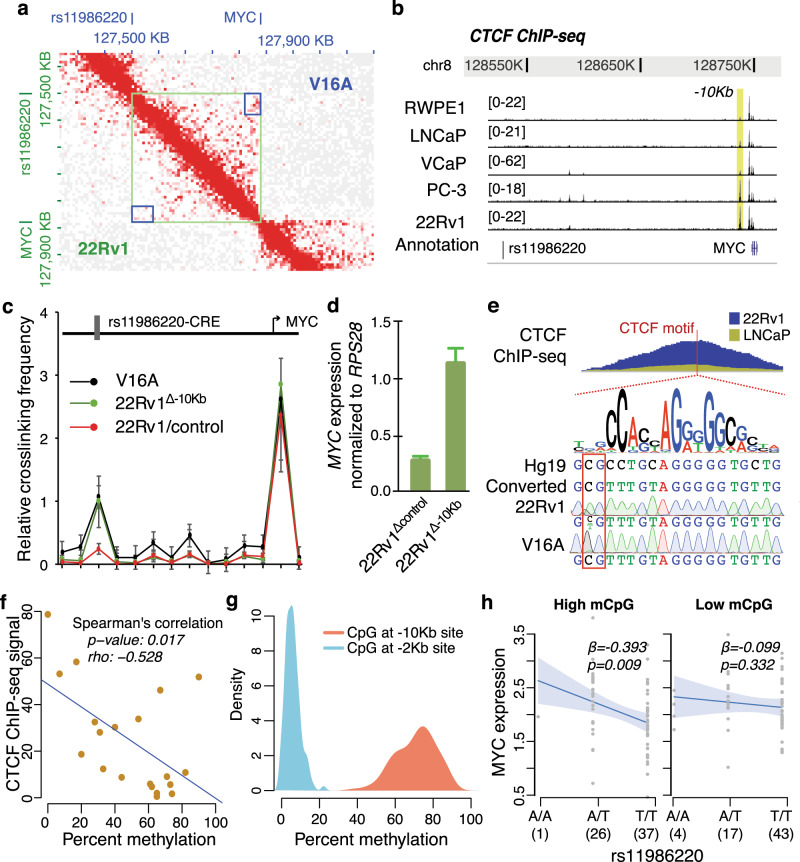
Methylation-dependent variable CTCF binding at –10 Kb locus regulates *MYC* eQTL. **a** Hi-C interaction map in 8q24.21 region in V16A (top-right triangle) and 22Rv1 cells (bottom-left triangle). The green square denotes the rs11986220-containing rCRE and *MYC*-promoter boundary and the blue square indicates the interaction points between these loci. **b** CTCF ChIP-seq signals in PCa cell lines between the rs1198220-containing rCRE and *MYC* promoter. The yellow bar denotes the CTCF binding site 10 Kb upstream of *MYC* promoter (–10 Kb site) which accumulates almost fourfold more CTCF deposition in 22Rv1 cells compared to LNCaP cells. **c** Interaction across chromatin regions between the rCRE and *MYC* promoter as determined by 3C assay. The data are shown in mean ± s.d. (*n* = 3). **d** Quantification of *MYC* transcripts by qPCR in 22Rv1 cells upon CRISPR/Cas9-mediated deletion of the –10 Kb CTCF site. Error bars denote standard error of mean (*n* = 2). **e** The top track indicates CTCF ChIP-seq profile in two cell lines and the position of CTCF binding motif (red bar). The motif logo is shown on the second track. The sequences shown are of the reference genome and bisulfite converted genome. The bottom two tracks show Sanger’s sequencing data upon bisulfite conversion in V16A and 22Rv1 cells. The red box denotes the differentially methylated CpG dinucleotide. **f** Correlation between the methylation level of this CpG and CTCF ChIP-seq signal at this locus in ENCODE cells. Each circle denotes a cell line and the blue line indicates the regression coefficient. See also Supplementary Fig. 4. **g** Distribution of methylation level of CpGs in –10 Kb and –2 Kb sites in 128 prostate tissues as determined by the whole-genome bisulfite sequencing. **h** Association between rs11986220 genotype and *MYC* expression in prostate tissues dichotomized by high (left panel) and low (right panel) level of methylation of the CpG in –10 Kb CTCF binding motif. The lines indicate the best fit for the regression models and the shaded areas indicate 95% confidence interval. The regression coefficient, β, and the *p* value are calculated using linear regression analysis. Source data are provided as a Source Data file.

Since promoter–CRE interaction is a spatial arrangement in 3D genome space, we performed Hi-C assay in both V16A and 22Rv1 cells to better understand the 3D genome architecture in these cell lines. The Hi-C data reveals that the rs11986220-containing rCRE interacts with *MYC* promoter in V16A cells but not in 22Rv1 cells (Fig. 4a). This suggests that 22Rv1 cells have a different 3D conformation than the V16A cells, which restricts the physical interaction between the rCRE and *MYC* promoter. A cell’s 3D chromatin structure is influenced by the protein CTCF^37,38^, and promoter–CRE interaction is usually encompassed by CTCF-mediated chromatin looping^39,40,65,66^. Several studies have reported that CTCF may form insulator loops blocking functions of nearby CREs^38,39,44–48,67,68^. Analyzing the CTCF ChIP-seq data in several PCa cell lines, we detected two CTCF-binding sites between the rs11986220-containing rCRE and *MYC* promoter—one is 10.4 Kb upstream (chr8:128737774–128738489; referred to as “–10 Kb” locus hereafter) and another 2.2 Kb upstream (chr8:128745980–128746790; referred to as “–2 Kb” locus hereafter) of *MYC* TSS (Fig. 4b). Between the two, CTCF binding is variable across PCa cell lines only at the –10 Kb locus, whereas 22Rv1 cells have almost fourfold higher binding than in LNCaP cells (Fig. 4b). The –2 Kb locus has been recently reported as a conserved and constitutive CTCF binding site with an enhancer-docking function to promote *MYC* expression^69^. We thus hypothesized that the higher CTCF binding at the –10 Kb locus in 22Rv1 cells blocks the *MYC* promoter–CRE interaction. To test this hypothesis, we generated two clonal variants of 22Rv1—22Rv1^Δ–10 Kb^ and 22Rv1^Δcontrol^, by expanding single-cell clones upon deletion of the –10 Kb locus and a neighboring control region, respectively, using CRISPR/Cas9 system. We then performed 3C experiments in these variants along with the V16A cells, and observed strong interaction between the rCRE and *MYC* promoter in V16A but not in 22Rv1^Δcontrol^ cells (Fig. 4c), in consistent with the Hi-C data (Fig. 4a). Depletion of the –10 Kb CTCF site resulted in strong interaction in 22Rv1^Δ–10 Kb^ similar to that observed in V16A cells (Fig. 4c). Consistently, *MYC* expression was induced by more than twofold in 22Rv1^Δ–10 Kb^ cells (Fig. 4d).

Thousands of CTCF-binding sites across the genome show variable binding affinity for CTCF, typically due to variable methylation levels of CpGs within the binding sites^70–72^. In consensus, hypermethylation of CpGs in CTCF-binding motif is correlated with lower CTCF binding and vice versa^70,73–75^. The DNA sequence at –10 Kb locus has a canonical CTCF binding motif near the summit of the CTCF ChIP-seq peak, and the first CpG in the motif is variably methylated in ENCODE cell lines (Fig. 4e and Supplementary Fig. 4a). We performed bisulfite conversion followed by Sanger sequencing and determined that this CpG is highly methylated in V16A, but lowly methylated in 22Rv1 cells (Fig. 4e). On the contrary, the methylation level at the –2 Kb site is consistently low, consistent with the constitutively high CTCF binding observed at this locus (Supplementary Fig. 4a). The effect of CpG methylation at the –10 Kb motif is evident by the inverse correlation observed between the methylation level and the CTCF ChIP-seq signals across the ENCODE cell lines (Spearman’s rho = –0.528; *p* = 0.017) (Fig. 4f and Supplementary Figs. 4b, c). To further validate, we coupled dCas9 with the methylating complex DNMT3A-3L and transfected 22Rv1 cells with sgRNAs targeting the –10 Kb and –2 Kb CTCF-binding sites. We observed that methylating the –2 Kb locus markedly decreases *MYC* expression consistent with a previous report^69^, but methylating the –10 Kb locus significantly increases *MYC* expression in 22Rv1 cells (Supplementary Fig. 4d).

### CTCF regulates the causal function of rs11986220

The SNP rs11986220 has a high risk OR for PCa among men from multiple ethnicities^13,35,36^. Despite the high prevalence of the risk allele A in the population, studies have failed to associate the SNP genotype with any gene in eQTL analyses in large cohorts (Supplementary Figs. 4e, f)^31,33,76^. Since the rs11986220-containing rCRE regulates *MYC* transcription only in absence of CTCF deposition at the –10 Kb locus (Fig. 4c, d), the effect of rs11986220 genotype on *MYC* expression may be masked by CTCF binding. To examine the effect of CTCF binding in SNP-gene association, we obtained the methylation (as a surrogate of CTCF occupancy), genotype, and RNA abundance data in 128 prostate tissues^77^. Similar as observed in the ENCODE data, methylation level at the –2 Kb site is constitutively low, while that of the –10 Kb site is highly variable (Fig. 4g). We then dichotomized the 128 samples based on the methylation level at –10 Kb motif into “High” and “Low” mCpG groups, and found the rs11986220 to be a strong eQTL for *MYC* (regression coefficient = 0.393; *p* value = 0.009) only in high mCpG subset but not in low mCpG subset (regression coefficient = −0.099; *p* value = 0.332) (Fig. 4h).

**Fig. 5:**
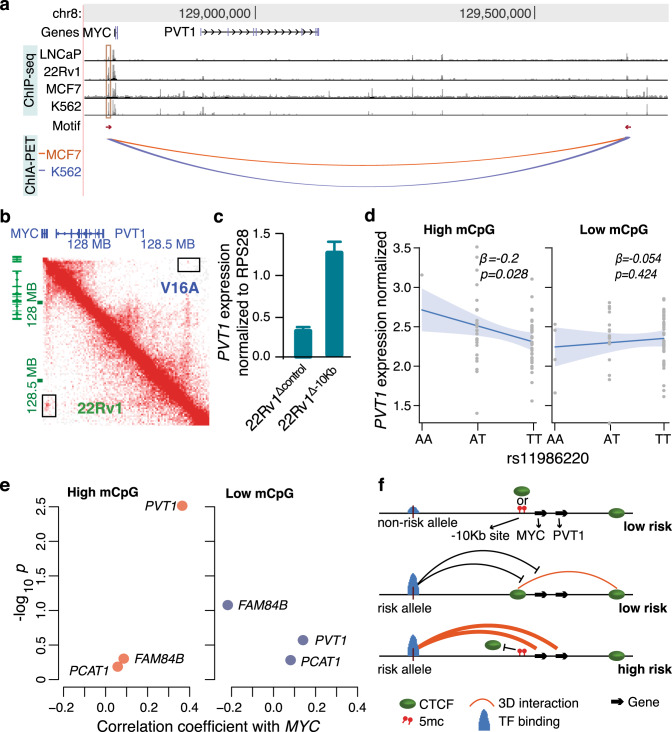
CTCF binding at –10 Kb site regulates both *MYC* and *PVT1*. **a** CTCF binding landscape downstream of *MYC*. The top four tracks show CTCF ChIP-seq signals in four cancer cell lines. The –10 Kb site is highlighted in red. The motif track shows a canonical CTCF-binding motif. The direction of the arrow indicates the orientation of the motif. The arcs show CTCF interactions between two CTCF binding sites in two cell lines as determined by ENCODE CTCF ChIA-PET data. **b** The Hi-C interaction map in V16A (top right triangle) and 22Rv1 (bottom left triangle) cells in this region. The interaction point in black rectangle denotes the interaction between two CTCF sites as shown in panel (**a**). **c** Quantification of *PVT1* expression in 22Rv1 cells upon deletion of –10 Kb site by CRISPR/Cas9. Error bars denote standard error of mean (*n* = 2). *P* value is estimated using *t* test. **d** Association between rs11986220 genotype and *PVT1* expression in prostate tissues dichotomized by high (left panel) and low (right panel) level of methylation of the CpG in CTCF binding motif at –10 Kb site. The lines indicate the best fit for the regression models and the shaded areas indicate 95% confidence interval. The regression coefficient, β, and the *p* value are calculated using linear regression analysis. **e** Pearson’s correlation coefficient between the expression of *MYC* and neighboring genes in prostate tissues dichotomized by the methylation level of the CpG. See Supplementary Fig. 5a for expression of neighboring genes. **f** Schematic of regulation of causal mechanism by methylation-dependent CTCF binding at –10 Kb site. Source data are provided as a Source Data file.

After confirming the enhancer-blocking function of CTCF at –10 Kb site, we further sought to investigate the chromatin plasticity mediated by this site. In ENCODE CTCF ChIA-PET data, the –10 Kb site interacts with another CTCF-binding site ~900 Kb downstream of *MYC* in MCF7 and K562 cells (Fig. 5a). Both MCF7 and K562 cells have high CTCF deposition at –10 Kb site comparable to that in 22Rv1 cells (Fig. 5a). Motif analysis reveals that these two CTCF sites have converging CTCF motifs that is often observed in interacting CTCF loci^38,43^ (Fig. 5a). Our Hi-C data also indicates that these two CTCF-binding sites indeed interact with each other in 22Rv1 cells, but not in V16A cells (Fig. 5b). Besides *MYC*, this cell line-specific insulator loop also includes the long noncoding RNA, *PVT1* (Fig. 5a). *PVT1* is another critical oncogene, which together with *MYC* drive tumorigenesis^78^. Deletion of the –10 Kb site also dramatically induces *PVT1* expression in 22Rv1 cells (Fig. 5c). Similar to *MYC*, rs11986220 genotype has a strong association with *PVT1* expression only in prostate tissue samples with high methylation at –10 Kb site (Fig. 5d). Amongst the expressed genes near *MYC* in prostate tissues, *MYC* expression is strongly correlated with only *PVT1* expression in tissue samples with high methylation, but the correlation is lost in tissue samples with low methylation at –10 Kb site **(**Fig. 5e and Supplementary Fig. 5a). These data suggest that the CTCF mediated looping blocks rs11986220-containing rCRE and disrupts the co-regulation of *MYC* and *PVT1*. The co-regulation of these two genes is critical in cancer predisposition, as the oncogenic function of *MYC* is dependent on *PVT1* expression and these two genes drive tumorigenesis synergistically^78,79^.

The enhancer-blocking insulator loop mediated by CTCF at the –10 Kb site thus mitigates the causal function of the PCa risk SNP rs11986220, and the risk conferred by rs11986220 is dependent on high methylation or low CTCF binding at the –10 Kb site (Fig. 5f). In fact, when analyzing the methylation level at –10 Kb site in five PCa patients carrying homozygous risk allele of rs11986220, we observed that the methylation level at –10 Kb motif is significantly higher in tumor compared to adjacent normal prostate tissues (*p* = 0.004; Student’s *t* test) (Supplementary Fig. 5b). Additionally, in our 3C interaction amplicons, we observed 1.5 fold higher enrichment of the risk allele A of rs11986220 in 22Rv1^Δ–10 Kb^ cells compared to 22Rv1^Δcontrol^ cells (Supplementary Fig. 5c). Altogether, this suggests that the risk allele of rs11986220 and higher methylation at –10 Kb function synergistically confer greater risk for PCa (Fig. 5f).

## Discussion

PCa genetic risk SNPs are enriched in noncoding CREs rather than in protein-coding regions^9,80^. It is challenging to systematically evaluate the importance of these rCREs in cancer biology and the clinic. Our study demonstrates that CRISPRi mediated loss-of-function screen of rCREs is an efficient approach to mitigate this challenge. We demonstrate that rCREs essential for PCa growth are characterized by higher H3K27ac modification. It is worth noting that many of the rCREs are not prostate specific and are also essential in lung cancer cell line A549. Identification and characterization of the target genes of these CREs will be of interest and warrant further investigation. Epigenomic profiling of prostate tissues and tumors is becoming prevalent^81–84^, which will be very useful to pinpoint essential rCREs for PCa predisposition and progression. By comparing risk scores of genetic predispositions with essentiality, we observed that the rCREs harboring stronger PCa genetic predispositions are more essential for PCa cell proliferation. In other words, genetic alteration in an essential rCRE puts the individual at a greater risk for PCa, further validating the importance of essentiality screens of rCREs. However, the CRISPRi technique is limited by the efficiency of individual sgRNAs, affecting the sensitivity of the assay. Furthermore, some cell lines such as LNCaP are challenging to generate a stable expression of dCas9-KRAB, which we failed to grow upon transduction. The rCRE screens can be further improved by using more densely tiled sgRNAs and more efficient fusion repressors. In addition, more comprehensive epigenomic profiling in screened cell lines will provide opportunities to better understand the biology underlying the essentiality.

We identified six rCREs that confer differential essentiality in V16A and 22Rv1 cells, five of which demonstrate differences in histone modification and transcription factor binding between the two cell lines. The underlying mechanisms warrant further investigation. The rs11986220-containing rCRE in the 8q24.21 region is specifically essential for the growth of V16A cells, but not the 22Rv1 cells. Our mechanistic analysis revealed that the differential essentiality of this rCRE is not because of differences in enhancer activity but enhancer-blocking CTCF binding at the –10 Kb locus. The –10 Kb site is about 8 Kb upstream of another *MYC*-regulating CTCF site^69^. These two CTCF sites together add a layer of complexity to an already convoluted regulatory mechanism of *MYC* expression, in that the CTCF at the –2 Kb site acts as an enhancer-docking site and induces *MYC* transcription, whereas the CTCF at the –10 Kb site acts as an enhancer-blocker and suppresses *MYC*. The *MYC*-inducing CTCF at the –2 Kb site is conserved across several tissues, constitutive, and does not vary across tissues^69^. In contrast, the CTCF at the –10 Kb site is regulated by variable methylation of CpG in the CTCF motif. Coincidently, introducing methylation to the CpG sites in the –2 Kb and –10 Kb loci by dCas9-DNMT3A-3L resulted in decreased and increased MYC expression, respectively. The CTCF-binding motif at the –10 Kb site does not contain any common genetic variant or somatic mutation, which indicates that the variability of the methylation may be epigenetically regulated and warrants further investigation.

The 8q24.21 region harbors multiple risk loci, which cumulatively account for 25% of familial risk in PCa^28^. As the most well-studied oncogene in this locus, *MYC* has always been speculated as to the causal gene in 8q24.21. Although a few studies have demonstrated physical interactions between rCREs and *MYC* promoter in prostate and other cancer types^33,34,85,86^, no association between risk SNPs genotypes and *MYC* expression has been observed^31–33,76^. Lack of *MYC* eQTL has thus been a long-standing dilemma in understanding the causal mechanisms in the 8q24.21 region. Our finding that the rCRE-*MYC* promoter interaction is dependent on 3D genome architecture suggests that the frequent looping observed in 8q24.21 may contribute to the failure in identifying *MYC* eQTLs in prostate and other cancer types. Indeed, when we dichotomize the prostate tissue samples into high and low methylation groups based on the methylation level (as a surrogate for CTCF binding) at the CpG in the –10 Kb CTCF motif, we detected a strong association between rs11986220 genotype and *MYC* expression specifically in the high methylation group. This indicates that eQTL analysis can be confounded by intervening enhancer-blocking CTCF looping, which aligns with the previous reports that SNP-gene associations are less common in presence of an intervening CTCF binding site^67,87,88^. Upon further investigation, we found out that the rs11986220-containing rCRE also regulates *PVT1* transcription in a similar manner, indicating that this rCRE is a common enhancer for both *MYC* and *PVT1*. A recent study identified *MYC* and *PVT1* promoters to compete for the same set of enhancers in MCF7 cells^89^. The CTCF–CTCF interaction spanning *MYC* and *PVT1* that we identified in 22Rv1 cells is also evident in MCF7 cells in publically available CTCF ChIA-PET data. Since the enhancers they compete for are all located in *PVT1* introns, it is likely that the competition occurs only when the enhancers upstream of the –10 Kb site are blocked by CTCF looping. Since the causal function of rs11986220 depends on the absence of CTCF at the –10 Kb site, the OR (1.6) conferred by the risk allele of rs11986220 is thus underestimated without considering the methylation status at the –10 Kb site. In addition, the co-activation of *MYC* and *PVT1* in populations with risk alleles at rs11986220 and high methylation at –10 Kb site may confer much greater risk, as knockin of both *MYC* and *PVT1*, but not each one alone, drives tumorigenesis in genetic mouse models^78^.

In summary, we report CRIPSRi screens of rCREs in PCa and identify a causal mechanism synergistically driven by a risk SNP and 3D genome architecture. This led to the identification of the long-sought *MYC* eQTL in 8q24.21 region specifically in populations with high methylation at the –10 Kb CTCF site. Considering methylation-regulated variable CTCF binding is prevalent in the human genome, we believe this is a common mechanism that may affect many other risk loci. In fact, no eQTL can be found for about 50% of PCa risk loci^90^. Furthermore, epigenome-wide association studies (EWAS) have identified methylation levels of thousands of individual CpG sites to be associated with cancer^91,92^. Many SNPs, both risk-associated and not, have been identified to be associated with methylation levels of CpG sites in prostate tumors^93^. The intricate interplay among genetic, expression, methylation, and 3D structure variations indicate that incorporating EWAS with GWAS may elucidate the causal mechanisms of many risk loci. Integration of multi-omics data has already been proposed to perform better as biomarkers^94,95^. Additionally, there is increasing evidence of inter-individual differential methylation of CpG sites in both humans and mice^96–98^. Inter-individual differential methylation levels of many CpG sites in internal tissues were found strongly correlated with methylation levels in blood^99^. This increases the possibility of detecting risk-associated methylation sites using non-invasive techniques, such as in blood or plasma cell-free DNA^100^. Together, our study unveils a direction to combine genetic with epigenetic risks, thus expected to lead to a paradigm shift in current approaches of predisposition assessment.

## Methods

### Target selection and tiling sgRNA design

The library rCREs were selected from our previous study (Supplementary Data 6)^9^. Briefly, 122 prostate cancer risk-associated tag SNPs and 5271 LD SNPs (*r*2> = 0.8) were identified in respective populations (Caucasian, African, and Asian). The 5271 LD SNPs (in 122 loci) overlap with 270 CREs (defined as DNase I hypersensitive regions in LNCaP cells). These DNase I hypersensitive sites larger than 400 bp were split into 400 bp windows. For positive controls, the promoter regions of critical genes were selected as 400 bp window centering the transcription start sites (TSSs) of genes. The negative controls were selected as DNaseI non-hypersensitive sites.

We developed a custom python tool, named sgTiler, to design tiling small guide RNAs (sgRNAs) targeting the rCREs and promoter regions. In brief, the tool first identifies all possible guide RNAs using the PAM sequence. Then the algorithm estimates the efficiency using criteria previously described^50–53^. The specificity is estimated using mapping to the entire genome, and the off-target potential is estimated by the number of off-target mapping and if mapped to exons or open chromatin regions. Finally, the tool optimizes the number of sgRNAs per CRE by declustering and selecting sgRNAs equidistantly spaced across the entirety of the targeted region. Details of the algorithm can be found in the preprint of the tool^101^.

### Cell lines

22Rv1 and A549 cell lines were obtained from the American Type Culture Collection (ATCC® CRL-2505 and ATCC® CCL-185) while HEK293FT cell line was obtained from ThermoFisher (R70007). The LNCaP-derived V16A cell line has been previously described^102^. A549, 22Rv1, and V16A cells were cultured in RPMI1640 medium with 10% FBS (Wisent) and 1% Penicillin and Streptomycin (450-201-EL, Wisent). 293FT cells were cultured in DMEM medium containing 10% FBS (080150, Wisent), L-glutamine (25030-081, ThermoFisher), and non-essential amino acids (11140–050, ThermoFisher) supplemented with 500 µg/mL Geneticin (4727894001, Sigma-Aldrich). All cells were cultured at 37° in 5% CO_2_. All cell lines were authenticated by STR and routinely tested for mycoplasma using the EZ-PCR mycoplasma Test Kit (20-700-20, Biological Industries).

### CRISPRi pooled screening, sequencing, and analysis

sgRNAs were synthesized as 73-mer oligonucleotides (CustomArray, USA), GAAAGGACGAAACACCGNNNNNNNNNNNNNNNNNNNNGTTTTAGAGCTAGAAATA GCAAGTTAAAATAAGGC (N’s denote the sgRNA 19–20 nucleotide target sequence) and amplified by PCR as a pool using the following primers: TAACTTGAAAGTATTTCGATTTCTTGGCTTTATATATCTTGTGGAAAGGACGAAACACCG (Forward) and ACTTTTTCAAGTTGATAACGGACTAGCCTTATTTTAACTTGCTATTTCTAGCTCTAAAAC (Reverse). The PCR product was purified and then cloned in the pLV hU6-sgRNA hUbC-dCas9-KRAB-T2a-Puro (gift from Charles Gersbach—Addgene plasmid # 71236, one vector system) using BsmBI (R0580S, NEB). Ligation was performed using the NEBuilder® HiFi DNA Assembly Cloning Kit (E5520S, NEB) and transformed into an electrocompetent strain (Cat. 11635018, Stbl4; ThermoFisher) to achieve ~300x coverage. Colonies were scraped off plates using LB and plasmid DNA was extracted (NA0310, Sigma GenEluteTM HP Plasmid Maxiprep Kit). The library was submitted for NGS to confirm adequate library representation of each sgRNA.

Library virus was generated in HEK293FT cells and each cell line was titrated with library virus to achieve a low MOI. The MOI was determined as previously described^103,104^. Briefly, MOI was determined by infecting ~5–6 million cells with varying amounts of library virus for 24 h, which were then split into media with or without puromycin (ThermoFisher; Cat. # A11138-03) for 48–72 h (A549, 3.5 µg/mL; 22Rv1, V16A, 3 µg/mL; LNCaP, 2.5 µg/mL). A ratio between these two populations was calculated to determine the infection efficiency to achieve a MOI of ~0.3. The amount of library virus was scaled up along with the number of cells to ensure that on average every sgRNA was represented in ~300 cells. For each screen, cells were split into triplicates every 3–4 days, and maintained at 300x coverage throughout the screen. Samples were collected in replicates (*n* = 2) on day 0 and day 16 post puromycin selection for genomic DNA analysis. sgRNA inserts were amplified by PCR as previously described^103^ and sequenced on an Illumina HiSeq 2500.

After sequencing, the fastq files were first converted to fasta files using a custom shell script. For each sample, a custom bowtie database was generated by the command bowtie-build in bowtie suite (version 1.1.2)^105^. The library sgRNAs were mapped against the database for each sample using bowtie with the parameter *v* = 0 and default values for other parameters. Since the functional core of a noncoding region is harder to predict, assessing essentiality of any CRE using the entire 400 bp window may reduce the sensitivity of the assay. To address this, each targeted region in the library was split into 100 bp sliding windows (50 bp offset) ensuring at least two sgRNAs targets a window. The differential sgRNA abundance was estimated using the “test” command in the tool MAGeCK^106^. The 100 bp window with the lowest *p* value in each CRE is treated as a representative of the essentiality of the CRE. For adjacent 400 bp windows, we merge all windows into the most essential window. The depletion score of each CRE is the “neg.score” as reported by MAGeCK^106^. For differential essentiality analysis, we applied a mean-shift outlier test (Bonferroni *p* < 0.1) based on Studentized residuals in linear regression. The test was performed using the outlierTest function in the R package “car” version 3.0.3 (Fox and Weisberg 2011). The depletion *p* values in V16A and 22Rv1 cells were combined using the R package EmpiricalBrownsMethod^107^.

### DepMap CRISPR-Cas9 screen data

The loss of function knock-out screens of thousands of genes for LNCaP and 22Rv1 cells were obtained from the Achilles DepMap GeCKO 19Q1 project^25^. For the A549 cells, Achilles DepMap Public 19Q3 data were used. For both datasets, gene_effect.csv files were downloaded from the DepMap portal. The ranking of all genes was visualized using R. In the DepMap project, the average dependency score of essential genes was set to −1; so closer to −1 more essential the gene is.

### Chromatin immunoprecipitation and sequencing

Chromatin immunoprecipitation (ChIP) assay was performed using V16A cells. Protein A (88845, ThermoFisher) and G (88847, ThermoFisher) Dynabeads were mixed at a 1:1 ratio, and preincubated with 6 ug H3K27ac antibody (ab4729) 3 h before immunoprecipitation. Cells were crosslinked by 1% formaldehyde for 10 min and then quenched with 125 mmol/L glycine. After cold PBS wash, the nuclear fraction was extracted and sonicated in a water bath sonicator (Diagenode bioruptor). Chromatin lysate was incubated with antibody-conjugated beads overnight. After washing and reverse crosslinking, DNA was purified by phenol–chloroform extraction and subjected to library preparation using the ThruPLEX DNA-seq Kit (R400428, Rubicon Genomics) according to the manufacturer’s protocol. Sequencing was performed at the Princess Margaret Genomics Centre. All ChIP-seq data were aligned against Hg19 using Bowtie2 version 2.0.5^105^ and the peaks were called using MACS2 version 2.0.10 ^108^ in its default setting.

### Epigenetic analysis

The H3K27ac, H3K4me1, H3K4me3, AR, FOXA1, and HOXB13 ChIP-seq signal data in LNCaP, 22Rv1, and A549 were obtained from Gene Expression Omnibus with accession IDs GSM1249448, GSM1145323, GSM969571, GSM1069682, GSM1410789, and GSE96652, respectively. All signal data were downloaded in the bigwig format. For each rCRE, the highest signal for each factor was extracted using the R package “rtracklayer” version 1.42.2^109^. The correlation between depletion score and ChIP-seq signals was estimated using the R function “cor.test” and visualized using the R package “corrplot” version 0.84. The H3K27ac ChIP-seq data for 22Rv1 and A549 cells were obtained from ENCODE portal with accession numbers ENCFF905QBL and ENCFF256RBI, respectively. Before performing the regression between depletion score and H3K27ac signal in cell line-specific manner, the distribution of H3K27ac signals was reverse normalized by the orderNorm function of the R package “bestNormalize” version 1.4.2^110^. The depletion scores were kept unmodified. The regression analysis was performed using the function “lm” in R.

### SNP and essentiality association

The SNPs associated with prostate cancer risk were obtained from GWAS Catalog (Accession ID EFO_0001663, downloaded on May 4th, 2019)^59^. If a rCRE harbors multiple GWAS-derived risk SNP within the 600 bp window, SNP with the highest odds ratio (OR) is retained as representative of that rCRE. The distribution of OR of SNPs in the library CRE was plotted using density function in R.

### Motif analysis

The positional weight matrix (PWM) for the CTCF motif was obtained from HOCOMOCO database (v10) using the R package “MotifDb” version 1.24.1. The motif was visualized using the R package “ggseqlogo” version 0.1. The DNA sequences of CTCF peaks were obtained using the function getSeq in the R package “Biostrings” version 2.50.2 and R dataset “BSgenome.Hsapiens.UCSC.hg38”. Motifs were scanned in any given chromatin sequence using the function matchPWM with at least 75% similarity score in both forward and reverse direction.

### Analysis of CTCF and methylation level at –10 Kb site

To check the variation of methylation level at –10 Kb and –2 Kb sites, methylation fractions of CpG sites processed from the whole genome bisulfite data were downloaded for 89 cell lines from the ENCODE portal^111,112^. For the correlation analysis between CTCF ChIP-seq signal and methylation level at –10 Kb site, total methylation fraction was obtained for the CpG at chr8:127725891 (GRCh38) which is located within the CTCF binding motif at –10 Kb site. In total, 21 out of the 89 cell lines with methylation data also had to match CTCF ChIP-seq data. The cell line EFO:0001196 had low read coverage at chr8:127725891 (total reads <5) hence was removed from subsequent analyses. The CTCF ChIP-seq signal (i.e., fold change over background) bigwig track for the 20 cell lines was downloaded from the ENCODE portal. The largest value for the ChIP-seq fold change over background was considered as the representative signal for each peak. The neighboring non-binding site for CTCF was randomly chosen as a site with no CTCF binding in PCa cell lines at chr8: 126,876,479–126,877,065 (GRCh38; Termed as NBS in Supplementary Fig. 4c). The correlation between the methylation and CTCF binding was calculated in R.

In ENCODE, 47 samples had matching CTCF ChIP-seq and RNA-seq data. The processed RNA-seq data for these samples were downloaded from the ENCODE portal. The correlation was calculated in R. All ENCODE data were downloaded from https://www.encodeproject.org/.

### eQTL analysis

To investigate the effect of CpG methylation at the –10 Kb CTCF site on *MYC* eQTL, the 128 prostate tissues were dichotomized by the median methylation level at this site. A regression analysis was performed using the *MYC* expression as dependent variable and genotype of rs11986220 using the lm function in R. Before performing the regression, the expression data were transformed to a normal distribution by Boxcox transformation with a lambda value of –0.1 using the R package “caret” version 6.0.84. The interaction terms between the genotype and methylation were plotted using the R package “effects” version 4.1.1.

### Epigenome editing by dCas9-3A-3L

22Rv1 cells were transfected with a dCas9-DNMT3A-3L (GFP) construct^69^ along with 3–5 guides that were cloned into pLKO5.sgRNA.EFS.tRFP (a kind gift of Benjamin Ebert, Addgene plasmid # 57823). Fourty-eight hours post transfection 30–50 K RFP + /GFP + cells were sorted and RNA extraction was performed followed by qPCR.

### DNA bisulfite conversion and targeted PCR

Genomic DNA from V16A and 22RV1 cells was isolated using DNeasy Blood & Tissue Kit (Cat. # 69504) and treated with RNAse A (EN0531, ThermoFIsher). Hundred nanograms of RNA-free gDNA was converted using the EZ DNA Methylation-Lightning Kit (ZYMO, D5030), and bisulfite-treated DNA was cleaned up using QIAquick PCR Purification Kit (QIAGEN 28106). PCR was performed using region-specific primers and ZymoTaqTM PreMix (ZYMO, E2003) to capture the CpG methylation status (Supplementary Data 5). PCR products were purified and submitted for Sanger sequencing using the reverse region-specific primer.

### RNA-sequencing

22Rv1 and V16A dCas9-KRAB stable cells were transduced individually with lentiviral particles containing two sgRNAs against the *MYC* enhancer, *MYC* promoter or a non-target region (*Luciferase* and *LacZ)*. Twenty-four hours post transduction, cells were selected with complete medium containing puromycin for 72 h. Total RNA was extracted using the RNeasy Mini Kit (74106, QIAGEN) according to manufacturer’s instructions. Following on-column DNase digestion, RNA-seq libraries were prepared using the TruSeq Stranded mRNA Library Preparation Kit (RS-122-2101, Illumina). TapeStation (Tape 2200, Agilent Technologies) was used to assess the quality of the libraries and sequencing was performed at the Princess Margaret Genomics Centre.

The raw sequencing data were mapped to human genome assembly Hg19 using TopHat2 version 2.1.0^113^ in its default setting. The reads per gene were counted using HTSeq version 0.7.2^114^ against refGene gene annotation^115^. The differential expression analyses were performed using DESeq2 package version 1.22.2 in R^116^. The differentially expressed genes were ranked in order of their fold change. The gene set enrichment analysis on the ranked gene list was performed using GSEA version 4.0.3 for the Hallmark gene set (H collection) in MSigDB^117,118^.

### Mouse xenograft experiments

All animal experiments were conducted in accordance with the study protocol 4714, which was approved by the University Health Network Research Ethics Board and Animal Care Committee. Four to six-week-old male NOD/SCID were obtained from Princess Margaret Cancer Centre Animal Research Centre (PMCC ARC) and housed under standard temperature, humidity, and timed lighting conditions mandated by the committee. Mice were randomly assigned across three experimental groups and used for xenograft experiments. In brief, a non-targeting sgRNA (*Luciferase)* or two individual sgRNAs targeting the rs11986220-CRE were transduced by lentivirus infection into V16A dCas9-KRAB stable cells. Following puromycin selection for 3 days, the cells were collected and washed with PBS. Cells were counted and one million cells were injected subcutaneously on the flank of each mouse in 0.1 mL of sterile PBS.

### CRISPRi sgRNA validation

sgRNA sequences were selected from the pooled library and cloned into the lentiGuide-Puro vector as previously described^104^. Lentiviral particles for each sgRNA were generated as mentioned above and transduced cells were selected with puromycin for 72 h. The sequences of the sgRNAs used in validation experiments are listed in Supplementary Data 5.

### Real-time PCR

Total RNA was purified with the RNeasy Mini Kit (QIAGEN, Cat. # 74106) and DNA was removed by performing on-column DNAse treatment (QIAGEN, Cat. # 79254). cDNA was reverse transcribed using the High Capacity cDNA Reverse Kit (4368814, Applied Biosystems). RNA expression was quantified using primers listed in Supplementary Data 5 along with PowerUp SYBR Green Master Mix (Applied Biosystems, Cat. # A25742). The CFX96 Touch Real-Time PCR Detection System (Bio-Rad) was employed to quantify RNA expression and all samples were normalized to RPS28. qRT-PCR was analyzed by the 2-^ΔΔ^CT method.

### Cell proliferation assays

Cellular proliferation assays were performed using methods previously described^103^. In brief, 2000–3000 cells per well of a 96 well were seeded (Falcon, Cat. # 353072) and imaged for 7 days using IncuCyte ZOOM live cell imaging system (Essen BioScience, MI USA). Cellular growth was calculated based on cell confluency (%).

### Lentiviral transduction and plasmids

The A549, V16A, and 22Rv1 dCas9-KRAB stable cell line was generated using the Lenti-dCas9-KRAB-blast plasmid (a gift from Gary Hon, Addgene plasmid # 89567). Lentiviral particles were generated in HEK293FT cells using the pMDG.2 and psPAX2 packaging plasmids (gift from Didier Trono—Addgene plasmids # 12259 and 12260). In brief, A549, V16A, and 22Rv1 cells were transduced for 24 h and selected with 10 or 5 µg/ml of blasticidin (450-190-WL, Wisent) for 5–7 days. Functional assays were performed to assess the activity of dCas9-KRAB by transducing stable cells with an sgRNA targeting the *MYC* promoter (See Supplementary Data 5). *MYC* expression levels were quantified by qPCR using primers listed in Supplementary Data 5. Viral particles containing sgRNAs targeting the *MYC* enhancer or non-targeting regions (*LacZ, Luciferase, NEG-1, and NEG-2*) were also generated using 293FT cells as previously mentioned.

### Generation of CTCF deleted variant in 22Rv1 using CRISPR/Cas9

Pairs of oligos were used for CRISPR/Cas9 mediated deletion of specific DNA fragments (See Supplementary Data 5). sgRNAs were cloned into the lentiCRISPRv2 (a gift from Feng Zhang, Addgene plasmid # 52961) and lentiCRISPRv2blast (gift from Brett Stringer, Addgene plasmid # 98293). Third-generation lentivirus vectors were used for packaging in 293T cell lines. The cells were trypsinized and seeded into 12-well plates, and 24 h later medium was replaced with low glucose DMEM containing 10% FBS, 0.1% penicillin, and streptomycin. Cells were transfected with specific constructs that have previously described^14^, pVSVG (envelope plasmid), pMDLg/pRRE (packaging plasmid), and pRSV-Rev (packaging plasmid) plasmids by Lipofectamine 2000 reagent (Cat. # L3000015, ThermoFisher). The medium was replaced 24 h post transfection and the medium containing viral particles was collected every 12 h. Lentivirus medium was filtered through 0.45 µm filters and snap freezing with liquid nitrogen. Target cells were seeded in six-well plates and transduced 16 h later with lentivirus-containing medium. Twenty-four hours post transduction, the medium was replaced with complete media containing 6 ug/mL blasticidin (450-190-WL, Wisent**)** and 3.5 ug/ml puromycin (ThermoFisher**)**. Single cells were obtained by serial dilution into 96-well plates and positive clones containing the deletion were examined by PCR followed by Sanger sequencing.

### Double deletions

22Rv1 clones deleted of the CTCF region were transduced with lentivirus containing pairs of sgRNAs against a control region or the rs11986220-CRE (backbone lentiCRISPRv2 and lentiCRISPRv2blast) for 24 h. Following this incubation, media was replaced with completed media and expanded for 4 days. Genomic DNA and RNA were extracted simultaneously using the AllPrep DNA/RNA Mini Kit (80204, Qiagen). Deletion efficiency was assessed by PCR using primers spanning regions upstream/downstream of the deleted regions, while RNA expression was assessed by qPCR following cDNA conversion (High Capacity cDNA Reverse Kit, ThermoFisher Cat. # 4368814).

### Quantitative analysis of chromosome conformation capture assays

3C experiments were performed using methods as previously described^119^. Briefly, ten million cells were trypsinized and resuspended in a 10% FBS/PBS buffer. Cells were fixed by 1% formaldehyde in 10 ml of 10% FBS/PBS buffer for 10 min at room temperature. The reaction was quenched with ice-cold glycine. Following centrifugation, the pellets were washed with cold PBS and re-suspended in a lysis buffer (10 Mm NaCl; 10 mM Tris-HCl, pH 7.5; 0.2% NP-40; 1x protease inhibitor). Nuclear extracts were obtained post centrifugation and *HindIII* (NEB, R0104S) was used for genomic DNA digestion. Digestion efficiency was assessed by SYBR-qPCR and only completely digested chromatin DNA was ligated using T4 DNA ligase. After reverse crosslinking, DNA fragments were purified by ethanol precipitation. The concentration of ligated DNA samples was measured by SYBR-qPCR and the samples were diluted to 100 ng/µL before running TaqMan qPCR. Each TaqMAN qPCR reaction contained 1 µl sample, 5 µl Quantitech probe PCR mix (QIAGEN), 1 µL 1.5 µM Taqman probe, 1 µL primers, and 2 µL water. Control samples include 14 *HindIII* sites and all DNA fragments were mixed together. The standard curve of each primer was generated by serial dilution of the control template and results were normalized to ERCC3 as control. All the primers for this experiment are listed in Supplementary Data 5.

### Hi-C experiment and analysis

HiC was performed using ARIMA-HiC kit (ARIMA) and the libraries were obtained with KAPA hyper preparation kit (KAPA) both using the instructions provided by ARIMA-HiC kit. Briefly, Cells were lysed in a lysis buffer and crosslinked with formaldehyde at 2% final concentration. Five micrograms of crosslinked DNA per sample in duplicates was digested and biotinylated with the provided pool of enzymes (enzymes A to D) in separate steps and the digested-biotinylated DNA was purified by using AMPure XP beads. DNA was size selected between 200–600 bp using AMPure XP beads. Provided Enrichment beads were used to enrich biotinylated DNA fragments and libraries were prepared by using Illumina TruSeq sequencing adapters (Illumina). The sequencing data was processed using the Hi-C Pro pipeline at it is default configuration^120^. The Arima-HiC cutsite file was generated using the tool digest_genome.py with the value of the parameter –*r* set as ^GATC G^AATC G^ATTC G^ACTC G^AGTC. Lastly, in order to prevent substantial unnecessary data loss the following modifications were made to the HiC-Pro configuration file.

LIGATION_SITE = GAATAATC,GAATACTC,GAATAGTC,GAATATTC,GAATGATC,GACTAATC,GACTACTC,GACTAGTC,GACTATTC,GACTGATC,GAGTAATC,GAGTACTC,GAGTAGTC,GAGTATTC,GAGTGATC,GATCAATC,GATCACTC,GATCAGTC,GATCATTC,GATCGATC,GATTAATC,GATTACTC,GATTAGTC,GATTATTC,GATTGATC

MIN_FRAG_SIZE = 10

MAX_FRAG_SIZE = 100,000

MIN_INSERT_SIZE = 100

MAX_INSERT_SIZE = 1000

### Statistical analysis

Throughout the study, continuous variables are presented using the median and interquartile range. Discrete variables are reported as the actual number or in percentages. All statistical analyses were performed in R programming language. For comparative analyses, a *p* ≤ 0.05 was considered significant unless stated otherwise. The differential abundance analyses, either for sgRNAs or mRNA molecules, were performed using negative binomial tests. Differences between two groups were estimated using two-tailed Student’s *t* test. Linear regression was performed to estimate the replicability of the CRISPRi screens across prostate cancer cell lines. Localized enrichment of essential CREs was estimated using Chi-squared test. The proliferation/tumor growth upon different treatments was compared using ANOVA test. An eQTL effect size and statistical significance were obtained from the *β* and *p* value as determined using linear regression analysis.

### Reporting summary

Further information on research design is available in the Nature Research Reporting Summary linked to this article.

## Supplementary information

Supplementary Information

Description of Additional Supplementary Files

Supplementary Data 1

Supplementary Data 2

Supplementary Data3

Supplementary Data 4

Supplementary Data 5

Supplementary Data 6

Reporting Summary

## Data Availability

Raw and processed sequencing data are deposited in the Gene Expression Omnibus (GEO) under the accession “GSE142811”. The H3K27ac ChIP-seq data for LNCaP and 22Rv1 cells were obtained from GEO with the accession IDs “GSM1249448” and “GSM2827407”, respectively. The H3K27ac ChIP-seq data for A549 cells were obtained from the ENCODE portal with the accession ID “ENCFF256RBI [https://www.encodeproject.org/experiments/ENCSR778NQS/]”. The H3K4me1, H3K27me3, AR, FOXA1, and HOXB13 ChIP-seq data were obtained from GEO with the accession IDs “GSM1145323”, “GSM969571”, “GSM1069682”, “GSM1410789”, and “GSM2537231”, respectively. The CTCF motif was obtained from the R package MotifDb (version 1.24.1) with the ID Hsapiens-HOCOMOCOv10-CTCFL_HUMAN.H10MO.A. The accession numbers of methylation and CTCF binding data from the ENCODE portal are listed in the Source Data. For the geneset enrichment analysis, the “H” collection was used from the MSigDB database (http://software.broadinstitute.org/gsea/msigdb/index.jsp). The DepMap data were obtained from https://ndownloader.figshare.com/files/16757666 and https://figshare.com/articles/DepMap_GeCKO_19Q1/7668407 for the DepMap Public 19Q3 and DepMap GeCKO 19Q1 libraries, respectively. CPC-GENE data obtained from the European Genome-phenome Archive with the accession number EGA: EGAS00001000900. In order to determine the association between the methylation level and CTCF binding at –10 Kb site, the WGBS data for the ENCODE samples UBERON:0001159, EFO:0001086, UBERON:0002046, UBERON:0002369, UBERON:0000992, EFO:0006711, EFO:0003072, EFO:0001187, UBERON:0001150, UBERON:0000473, UBERON:0000945, UBERON:0002106, CL:0000182, UBERON:0008952, UBERON:0002367, UBERON:0004264, UBERON:0001323, EFO:0002791, EFO:0002067, and CL:0002327 were downloaded from https://www.encodeproject.org/files/ENCFF157POM/@@download/ENCFF157POM.bed.gz, https://www.encodeproject.org/files/ENCFF005TID/@@download/ENCFF005TID.bed.gz, https://www.encodeproject.org/files/ENCFF497IYX/@@download/ENCFF497IYX.bed.gz, https://www.encodeproject.org/files/ENCFF216DJL/@@download/ENCFF216DJL.bed.gz, https://www.encodeproject.org/files/ENCFF189WPY/@@download/ENCFF189WPY.bed.gz, https://www.encodeproject.org/files/ENCFF782JXT/@@download/ENCFF782JXT.bed.gz, https://www.encodeproject.org/files/ENCFF940XWW/@@download/ENCFF940XWW.bed.gz, https://www.encodeproject.org/files/ENCFF064GJQ/@@download/ENCFF064GJQ.bed.gz, https://www.encodeproject.org/files/ENCFF699RBP/@@download/ENCFF699RBP.bed.gz, https://www.encodeproject.org/files/ENCFF715DMX/@@download/ENCFF715DMX.bed.gz, https://www.encodeproject.org/files/ENCFF844EFX/@@download/ENCFF844EFX.bed.gz, https://www.encodeproject.org/files/ENCFF333OHK/@@download/ENCFF333OHK.bed.gz, https://www.encodeproject.org/files/ENCFF366UWF/@@download/ENCFF366UWF.bed.gz, https://www.encodeproject.org/files/ENCFF842MHJ/@@download/ENCFF842MHJ.bed.gz, https://www.encodeproject.org/files/ENCFF027KTR/@@download/ENCFF027KTR.bed.gz, https://www.encodeproject.org/files/ENCFF219GCQ/@@download/ENCFF219GCQ.bed.gz, https://www.encodeproject.org/files/ENCFF843SYR/@@download/ENCFF843SYR.bed.gz, https://www.encodeproject.org/files/ENCFF804NTQ/@@download/ENCFF804NTQ.bed.gz, https://www.encodeproject.org/files/ENCFF867JRG/@@download/ENCFF867JRG.bed.gz, and https://www.encodeproject.org/files/ENCFF699GKH/@@download/ENCFF699GKH.bed.gz, respectively. The CTCF ChIP-seq signal tracks for the same set of samples were downloaded from https://www.encodeproject.org/files/ENCFF402URE/@@download/ENCFF402URE.bigWig, https://www.encodeproject.org/files/ENCFF848ZIL/@@download/ENCFF848ZIL.bigWig, https://www.encodeproject.org/files/ENCFF551SXN/@@download/ENCFF551SXN.bigWig, https://www.encodeproject.org/files/ENCFF858KCB/@@download/ENCFF858KCB.bigWig, https://www.encodeproject.org/files/ENCFF500IGC/@@download/ENCFF500IGC.bigWig, https://www.encodeproject.org/files/ENCFF932OWO/@@download/ENCFF932OWO.bigWig, https://www.encodeproject.org/files/ENCFF698JTY/@@download/ENCFF698JTY.bigWig, https://www.encodeproject.org/files/ENCFF894ZLN/@@download/ENCFF894ZLN.bigWig, https://www.encodeproject.org/files/ENCFF744UAL/@@download/ENCFF744UAL.bigWig, https://www.encodeproject.org/files/ENCFF599OCP/@@download/ENCFF599OCP.bigWig, https://www.encodeproject.org/files/ENCFF913XZA/@@download/ENCFF913XZA.bigWig, https://www.encodeproject.org/files/ENCFF603HTW/@@download/ENCFF603HTW.bigWig, https://www.encodeproject.org/files/ENCFF350UER/@@download/ENCFF350UER.bigWig, https://www.encodeproject.org/files/ENCFF306NUN/@@download/ENCFF306NUN.bigWig, https://www.encodeproject.org/files/ENCFF686GDH/@@download/ENCFF686GDH.bigWig, https://www.encodeproject.org/files/ENCFF645MYR/@@download/ENCFF645MYR.bigWig, https://www.encodeproject.org/files/ENCFF497MQF/@@download/ENCFF497MQF.bigWig, https://www.encodeproject.org/files/ENCFF991GMN/@@download/ENCFF991GMN.bigWig, https://www.encodeproject.org/files/ENCFF799LZZ/@@download/ENCFF799LZZ.bigWig, and https://www.encodeproject.org/files/ENCFF592HCJ/@@download/ENCFF592HCJ.bigWig. All other relevant data supporting the key findings of this study are available within the article and its Supplementary Information files or from the corresponding author upon reasonable request. A reporting summary for this article is available as a Supplementary Information file. Source data are provided with this paper.

## Source data

Source Data

## Code availability

The codes to design the tiling sgRNA library is available at https://github.com/HansenHeLab/sgTiler^121^.
