# Supplementary material for: CRISPRi screens reveal a DNA methylation-mediated 3D genome dependent causal mechanism in prostate cancer
Source: Nat Commun. 2021 Mar 19;12:1781. doi: 10.1038/s41467-021-21867-0 (PMC7979745; doi:10.1038/s41467-021-21867-0)
Supplement: Supplementary file 1 — Supplementary Information [file 41467_2021_21867_MOESM1_ESM.pdf]

CRISPRi screens reveal a DNA methylation-mediated 3D genome dependent causal mechanism in prostate cancer

Supplementary materials

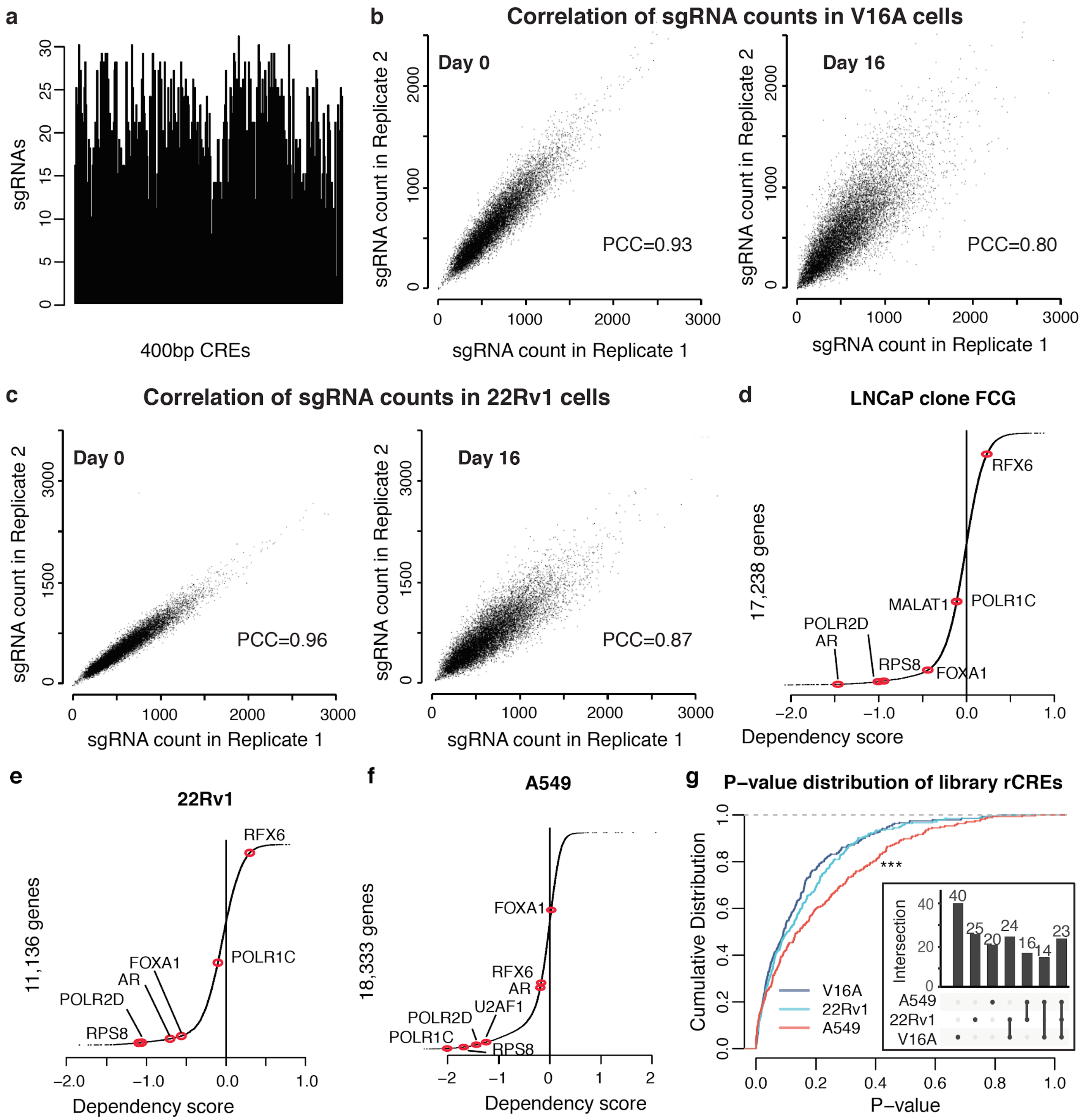

Supplementary Figure 1: Overview of CRISPRi screen library, related to Figure 1. a) Number of sgRNAs in each of the rCREs. **b,c)** Correlation of sgRNA counts between replicates at two time points in V16A and 22Rv1 cells. PCC, Pearson's correlation coefficient. **d)** Rank of essentiality effects of genes upon CRISPR knock-out in LNCaP, **(e)** 22Rv1, and **(f)** A549 cells as determined in the Achilles DepMap project. The genes whose promoters are targeted in this study are labeled. Lower the dependency score, higher the essentiality for cell growth. **g)** Empirical cumulative distribution of p-value of depletion in three cell lines. ***, p≤0.0001 in Kolmogorov Smirnov test. The barplot in the inset indicates the intersection of CREs with depletion p-value ≤ 0.05 in the three cell lines.

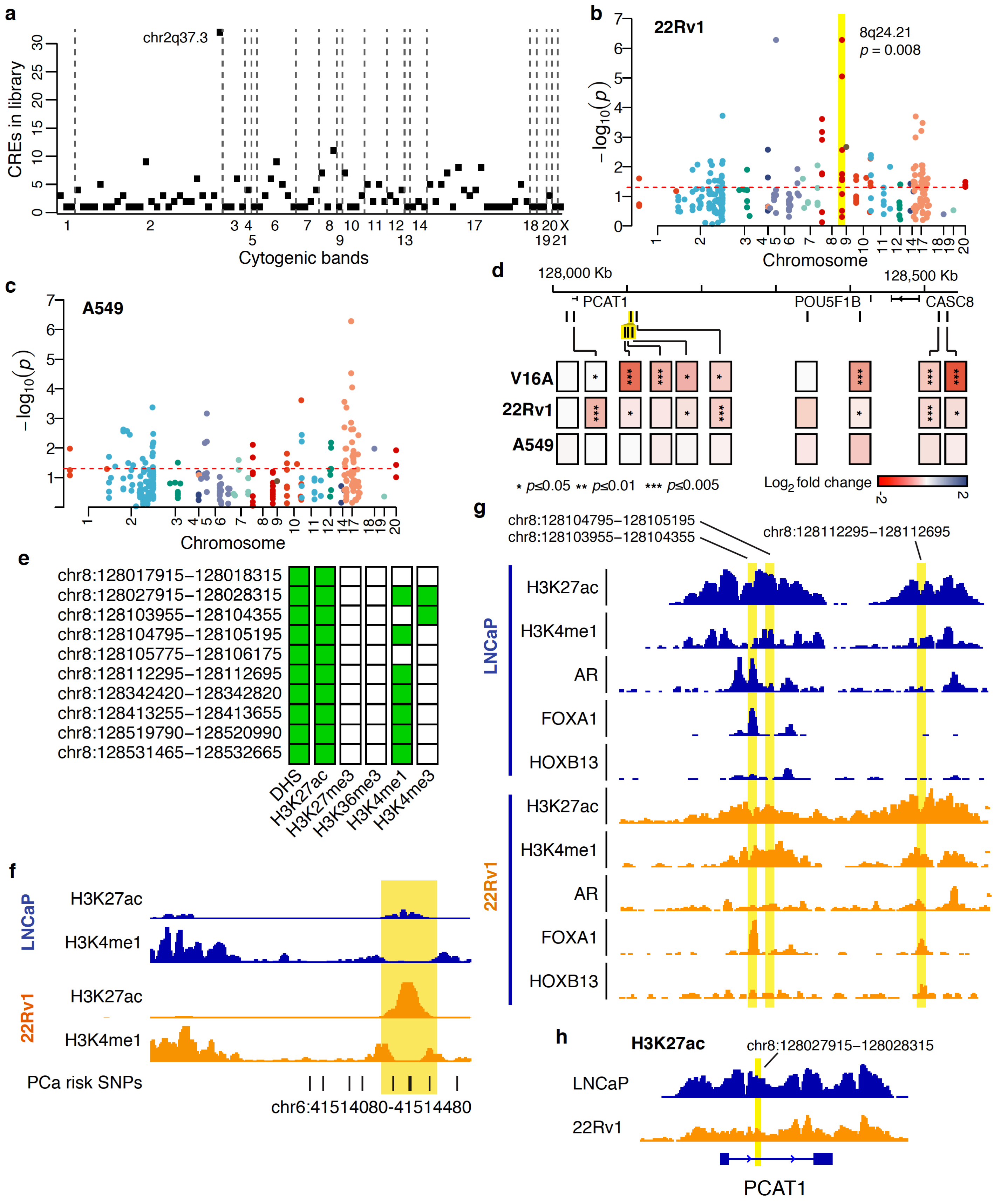
a
chr2q37.3
CREs in library
Cytogenic bands
b
22Rv1
8q24.21
p = 0.008
−log10(p)
Chromosome
c
A549
−log10(p)
Chromosome
d
128,000 Kb
128,500 Kb
PCAT1
POU5F1B
CASC8
V16A
22Rv1
A549
* p≤0.05 ** p≤0.01 *** p≤0.005
Log2 fold change
e
chr8:128017915–128018315
chr8:128027915–128028315
chr8:128103955–128104355
chr8:128104795–128105195
chr8:128105775–128106175
chr8:128112295–128112695
chr8:128342420–128342820
chr8:128413255–128413655
chr8:128519790–128520990
chr8:128531465–128532665
DHS
H3K27ac
H3K27me3
H3K36me3
H3K4me1
H3K4me3
f
LNCaP
H3K27ac
H3K4me1
22Rv1
H3K27ac
H3K4me1
PCa risk SNPs
chr6:41514080-41514480
g
chr8:128104795–128105195
chr8:128103955–128104355
chr8:128112295–128112695
LNCaP
H3K27ac
H3K4me1
AR
FOXA1
HOXB13
22Rv1
H3K27ac
H3K4me1
AR
FOXA1
HOXB13
h
H3K27ac
chr8:128027915–128028315
LNCaP
22Rv1
PCAT1

Supplementary Figure 2: Essential rCREs are enriched in 8q24.21, related to Figure 2. a) Distribution of library CREs in the genome. **b,c)** Manhattan plot of essentiality of rCREs in 22Rv1 and A549 cells. The size of the points is related with the depletion fold. The p-values were estimated using Chi-squared test. **d)** Schematic of rCRE positions in the gene desert region of 8q24.21 region. Vertical bars locate the rCREs. The colors in the box denote the fold depletion. P-value is calculated by a negative binomial test using MAGeCK (see Methods). **e)** Histone marks in rCREs in the gene desert region of 8q24.21. Green indicates presence of histone modification as determined by peak calling in respective ChIP-seq data. **f)** ChIP-seq signals of histone marks in LNCaP and 22Rv1 cells in chr6:41514080-41514480. **g)** ChIP-seq signals of histone marks and transcription factors in LNCaP and 22Rv1 cells in 8q24.21 region spanning three library rCREs. **h)** ChIP-seq signals of H3K27ac marks near PCAT1 region. The region highlighted in yellow has a higher depletion score in 22Rv1 than V16A cells.

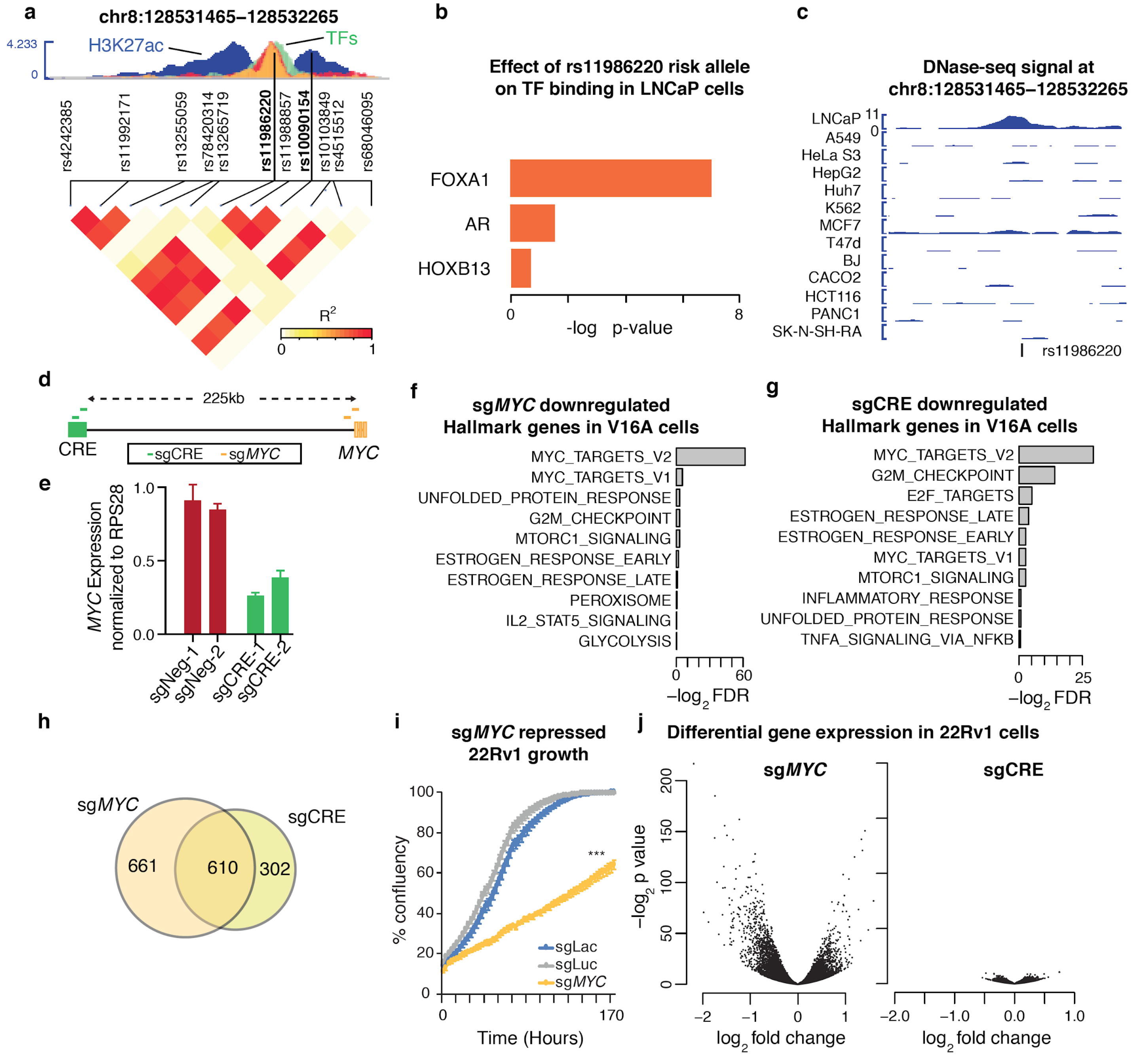

Supplementary Figure 3: The rs11986220-containing rCRE regulates *MYC* in V16a cells, related to Figure 3. a) The differential rCRE in V16A cells is overlapped with two PCa-associated risk SNPs marked in bold letters. The haplotype map indicates the linkage disequilibrium among the SNPs in 1000 Genome Project phase III European population. The top track demonstrates the ChIP-seq signals of H3K27ac modification (blue) and overlapped signal of AR (orange), FOXA1 (green) and HOXB13 (red) binding. **b)** Effect of the rs11986220 on binding of three transcription factors in LNCaP cells as determined by IGR (see Methods). **c)** DNase-seq signal in ENCODE cancer cell lines overlapping rs11986220-containing rCRE. **d)** Schematic of sgRNAs targeting the rCRE and *MYC* promoter, related to Figure 3c-g. **e)** *MYC* expression by qPCR upon suppression of rCRE by independent sgRNAs. The error bars denote standard error of mean (n=2). Source data are provided as a Source Data file. **f)** Top

enriched gene families upon repression of *MYC* promoter or **(g)** rCRE in V16 cells. P-values were estimated using geneset enrichment analysis (see Methods) and the False Discovery Rate was calculated by adjusting the p-values for multiple correction. **h)** Venn diagram indicating the number of differentially expressed genes upon suppression of rs11986220-containing CRE (sgCRE) or MYC promoter (sgMYC) in V16A cells. **i)** Cellular proliferation assay of 22Rv1 cells upon repression of controls (sgLac or sgLuc) and *MYC* promoter by sgRNA guided dCas9-KRAB complex. The p value is estimated using ANOVA test. **j)** Volcano plot of differentially expressed genes in 22Rv1 cells upon repression of *MYC* promoter or the rCRE.

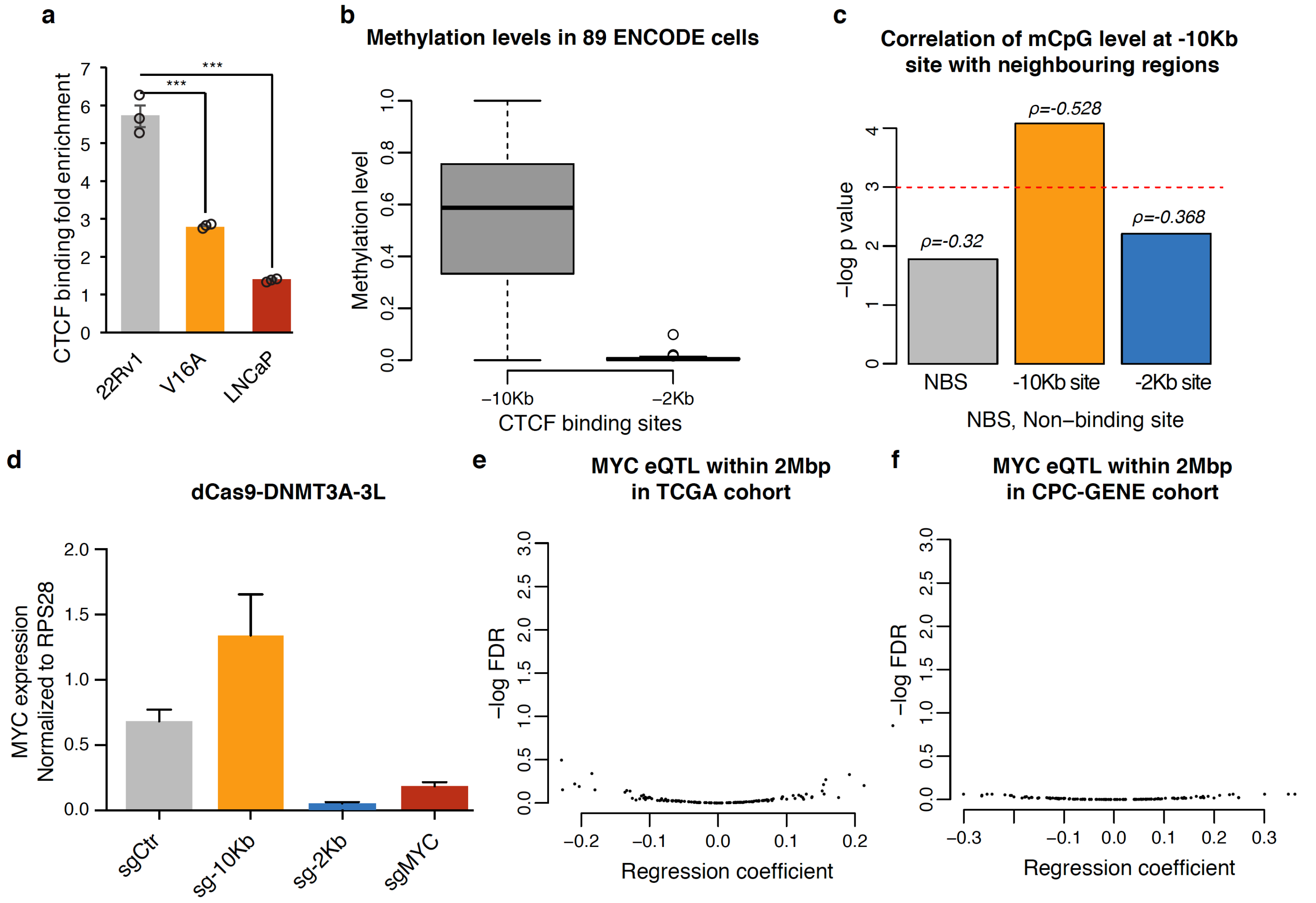

Supplementary Figure 4: CTCF at -10Kb site regulated by CpG methylation in motif, related to Figure 4. a) CTCF binding in three prostate cancer cell lines as determined by ChIP-qPCR. The error bar indicates standard error (n=3). The p-value is estimated by two-sided unpaired Student's t-test. ***, $p \leq 0.01$. **b)** Box and whiskers plot depicting the methylation levels at -2Kb and -10Kb sites across cohort (n=89). The upper and lower limits of the box denote the third and first quartile, respectively. The black horizontal line denotes the median, the whiskers indicate the furthest data points within 1.5 times of the interquartile range and the black circles indicate outliers. **c)** Spearman's correlation between methylation at -10Kb CTCF motif and CTCF ChIP-seq signals in ENCODE cell lines. NBS is selected

as the nearest CTCF non-binding site. **d)** *MYC* expression by qPCR upon targeted methylation of a control negative region, -10Kb site, -2Kb site and *MYC* promoter. The error bar indicates standard error (n=2). P value is estimated by Student's t-test. ***, p ≤ 0.01. **e)** eQTL analysis between *MYC* expression and genotype of SNPs within 2Mbp in TCGA PRAD and **(f)** CPC-GENE cohorts. Source data are provided as a Source Data file.

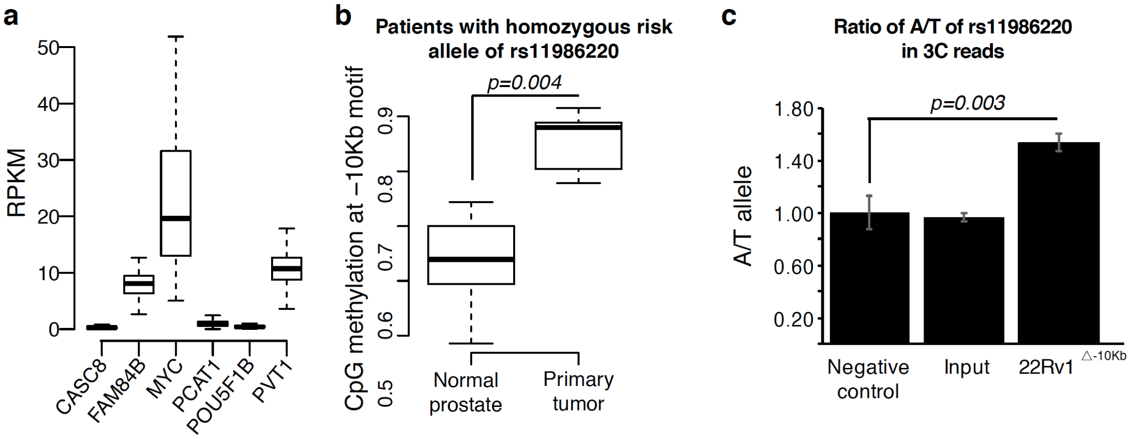

Supplementary Figure 5: Expression of 8q24.21 genes and methylation in 128 prostate tissues, related to Figure 5. a) Expression of 8q24.21 genes in 128 prostate tissues. *CASC8* and *POU5F1B* gene is very lowly expressed and hence removed from downstream analysis in Figure 5e. The upper and lower limits of the box denote the third and first quartile, respectively. The black horizontal line denotes the median and the whiskers indicate the furthest data points within 1.5 times of the interquartile range. **b)** The methylation level of CpG at -10Kb CTCF motif in normal prostate and tumor tissues in five patients with homozygous risk allele of rs11986220. The upper and lower limits of the box denote the third and first quartile, respectively. The black horizontal line denotes the median and the whiskers indicate the furthest data points within 1.5 times of the interquartile range. The p-value is estimated using Student's unpaired t-test. **c**) Ratio of A to T allele of rs11986220 in 3C ligated amplicons between rs11986220-containing CRE and MYC promoter region. The error bars indicate s.d. (n=3). The p-value is estimated using Student's unpaired t-test. Source data are provided as a Source Data file.
