# Supplementary material for: CRISPRi screens reveal a DNA methylation-mediated 3D genome dependent causal mechanism in prostate cancer
Source: Nat Commun. 2021 Mar 19;12:1781. doi: 10.1038/s41467-021-21867-0 (PMC7979745; doi:10.1038/s41467-021-21867-0)
Supplement: Supplementary file 2 — Description of Additional Supplementary Files [file 41467_2021_21867_MOESM2_ESM.pdf]

Description of Additional Supplementary Files

Supplementary Data 1: sgRNA sequences of the CRISPRi screen library.

Supplementary Data 2: MAGeCK output of CRISPRi screen in V16A cells.

Supplementary Data 3: MAGeCK output of CRISPRi screen in 22Rv1 cells.

Supplementary Data 4: MAGeCK output of CRISPRi screen in A549 cells.

Supplementary Data 5: Primers sequences used in this study.

Supplementary Data 6: List of ldSNPs and rCREs included in the CRISPRi screen library.
