# Supplementary material for: CRISPRi screens reveal a DNA methylation-mediated 3D genome dependent causal mechanism in prostate cancer
Source: Nat Commun. 2021 Mar 19;12:1781. doi: 10.1038/s41467-021-21867-0 (PMC7979745; doi:10.1038/s41467-021-21867-0)
Supplement: Supplementary file 9 — Reporting Summary [file 41467_2021_21867_MOESM9_ESM.pdf]

nature research

Corresponding author(s): Housheng Hansen He & Gonghong Wei & Shancheng Ren

Last updated by author(s): Dec 17, 2020

Reporting Summary

Nature Research wishes to improve the reproducibility of the work that we publish. This form provides structure for consistency and transparency in reporting. For further information on Nature Research policies, see our Editorial Policies and the Editorial Policy Checklist.

Statistics

For all statistical analyses, confirm that the following items are present in the figure legend, table legend, main text, or Methods section.

n/a	Confirmed	
☐	☒	The exact sample size (*n*) for each experimental group/condition, given as a discrete number and unit of measurement
☐	☒	A statement on whether measurements were taken from distinct samples or whether the same sample was measured repeatedly
☐	☒	The statistical test(s) used AND whether they are one- or two-sided<br>*Only common tests should be described solely by name; describe more complex techniques in the Methods section.*
☐	☒	A description of all covariates tested
☐	☒	A description of any assumptions or corrections, such as tests of normality and adjustment for multiple comparisons
☐	☒	A full description of the statistical parameters including central tendency (e.g. means) or other basic estimates (e.g. regression coefficient) AND variation (e.g. standard deviation) or associated estimates of uncertainty (e.g. confidence intervals)
☐	☒	For null hypothesis testing, the test statistic (e.g. *F*, *t*, *r*) with confidence intervals, effect sizes, degrees of freedom and *P* value noted<br>*Give P values as exact values whenever suitable.*
☒	☐	For Bayesian analysis, information on the choice of priors and Markov chain Monte Carlo settings
☒	☐	For hierarchical and complex designs, identification of the appropriate level for tests and full reporting of outcomes
☐	☒	Estimates of effect sizes (e.g. Cohen's *d*, Pearson's *r*), indicating how they were calculated

Our web collection on statistics for biologists contains articles on many of the points above.

Software and code

Policy information about availability of computer code

Data collection	No softwares were used for data collection
Data analysis	The CRISPRi screen was processed using Bowtie (v.1.1.2) and MAGeCK (v0.5.4). The RNA-seq data were processed using TopHat2 (v2.1.0), HTSeq-count (v0.7.2) and DESeq2 (v1.22.2). Geneset enrichment analysis was performed using GSEA (4.0.3). The data are analyzed in R (4.0.2) using packages car (3.0.3), rtracklayer (1.42.2), corrplot (0.84), bestNormalize (1.4.2), MotifDb (1.24.1), ggseqlogo (0.1), Biostrings (2.50.2), caret (6.0-84), effects (4.1-1).

For manuscripts utilizing custom algorithms or software that are central to the research but not yet described in published literature, software must be made available to editors and reviewers. We strongly encourage code deposition in a community repository (e.g. GitHub). See the Nature Research guidelines for submitting code & software for further information.

Data

Policy information about availability of data

All manuscripts must include a data availability statement. This statement should provide the following information, where applicable:

- Accession codes, unique identifiers, or web links for publicly available datasets
- A list of figures that have associated raw data
- A description of any restrictions on data availability

Raw and processed sequencing data are deposited in the Gene Expression Omnibus (GEO) under the accession GSE142811 (https://www.ncbi.nlm.nih.gov/geo/query/acc.cgi?acc=GSE142811). Source data are provided with this paper.
The H3K27ac ChIP-seq data for LNCaP and 22Rv1 cells were obtained from GEO with the accession IDs GSM1249448 and GSM2827407, respectively. The K3K27ac ChIP-seq data for A549 cells was obtained from the ENCODE portal with the accession ID ENCFF256RBI. The H3K4me1, H3K27me3, AR, FOXA1 and HOXB13 ChIP-seq data were obtained from GEO with the accession IDs GSM1145323, GSM969571, GSM1069682, GSM1410789 and GSM2537231, respectively. The CTCF motif was

obtained from the R package MotifDb (version 1.24.1) with the ID Hsapiens-HOCOMOCOv10-CTCFL_HUMAN.H10MO.A. The accession numbers of methylation and CTCF binding data from the ENCODE portal are listed in the Source Data. For the geneset enrichment analysis, the "H" collection was used from the MSigDB database (http://software.broadinstitute.org/gsea/msigdb/index.jsp). The DepMap data were obtained from https://ndownloader.figshare.com/files/16757666 and https://figshare.com/articles/DepMap_GeCKO_19Q1/7668407 for the DepMap Public 19Q3 and DepMap GeCKO 19Q1 libraries, respectively. CPC-GENE data obtained from the European Genome-phenome Archive with the accession number EGA: EGAS00001000900.

Field-specific reporting

Please select the one below that is the best fit for your research. If you are not sure, read the appropriate sections before making your selection.

☒ Life sciences ☐ Behavioural & social sciences ☐ Ecological, evolutionary & environmental sciences

For a reference copy of the document with all sections, see nature.com/documents/nr-reporting-summary-flat.pdf

Life sciences study design

All studies must disclose on these points even when the disclosure is negative.

Sample size	CRISPRi screen were done using two replicates for each time point for three different cell lines. RNA-seq were performed with the sample size of two for each cell line at each condition. Since these are in-vitro analyses using cell line models, two replicates are typically sufficient to address the variability.
Data exclusions	Whole genome bisulfite sequencing data of one ENCODE cell line was removed from subsequent analyses due to low read coverage (less than 5 reads). The cutoff was determined arbitrarily based upon the distribution of read coverage across all ENCODE WGBS data.
Replication	For each experiment, all attempts at replication were successful. Experiments were performed at least 2-3 times.
Randomization	Randomization not deemed essential for this study because the fundamental findings were made using cell lines and no case/control study was performed.
Blinding	Blinding not deemed essential for this study because the fundamental findings were made using cell lines nd no case/control study was performed.

Reporting for specific materials, systems and methods

We require information from authors about some types of materials, experimental systems and methods used in many studies. Here, indicate whether each material, system or method listed is relevant to your study. If you are not sure if a list item applies to your research, read the appropriate section before selecting a response.

Materials & experimental systems

n/a	Involved in the study	
☐	☒	Antibodies
☐	☒	Eukaryotic cell lines
☒	☐	Palaeontology and archaeology
☐	☒	Animals and other organisms
☒	☐	Human research participants
☒	☐	Clinical data
☒	☐	Dual use research of concern

Methods

n/a	Involved in the study	
☐	☒	ChIP-seq
☒	☐	Flow cytometry
☒	☐	MRI-based neuroimaging

Antibodies

Antibodies used	6ug of ab4729 (Abcam) for H3K27AC ChIP in V16A cells
Validation	Mumbach MR, Satpathy AT, Boyle EA, et al. Enhancer connectome in primary human cells identifies target genes of disease-associated DNA elements. Nat Genet. 2017;49(11):1602-1612. doi:10.1038/ng.3963

Eukaryotic cell lines

Policy information about cell lines

Cell line source(s)	22Rv1 and A549 cell lines were obtained from the American Type Culture Collection (ATCC® CRL-2505 and ATCC® CCL-185) while HEK293FT cell line was obtained from ThermoFisher (R70007). The LNCaP-derived V16A cell line has been previously described (Bishop et al. Cancer Discovery (2017).
Authentication	All cell lines were authenticated by STR using Geneprint10 panel system (TCAG, Canada).

Mycoplasma contamination	Cell lines were routinely tested for mycoplasma using the EZ-PCR mycoplasma Test Kit (20-700-20, Biological Industries). Cell lines used for experimental studies were negative for mycoplasma
Commonly misidentified lines (See ICLAC register)	No commonly misidentified cell lines were used in the study,

Animals and other organisms

Policy information about studies involving animals; ARRIVE guidelines recommended for reporting animal research

Laboratory animals	Four to six-week old male NOD/SCID were obtained from Princess Margaret Cancer Centre Animal Research Centre and housed under 20-22C temperature, 45-60% humidity, and 12:12 hours dark/light cycle conditions as mandated by the committee.
Wild animals	No wild animals were used in the study
Field-collected samples	No field collected samples were used in the study
Ethics oversight	All animal experiments were conducted in accordance with the University Health Network and Animal Care Committee.

Note that full information on the approval of the study protocol must also be provided in the manuscript.

ChIP-seq

Data deposition

- [x] Confirm that both raw and final processed data have been deposited in a public database such as GEO.
- [x] Confirm that you have deposited or provided access to graph files (e.g. BED files) for the called peaks.

Data access links *May remain private before publication.*	https://www.ncbi.nlm.nih.gov/geo/query/acc.cgi?acc= GSE14281
Files in database submission	GSM4240450_V16A-HICHIP_S1_L001_R1_001.fastq.bam_SPMR_peaks.narrowPeak.gz
Genome browser session (e.g. UCSC)	Not available

Methodology

Replicates	1
Sequencing depth	28.1M reads, 75bp length, single-end
Antibodies	ab4729
Peak calling parameters	MACS2 with default parameters
Data quality	The ChIP-seq data were visualized in IGV. A total of 25,743 peaks were identified over q-value of 0.05. A total of 4,251 peaks had a fold change of 5 or higher.
Software	Bowtie2 and MACS2
